# Dysregulated Provision of Oxidisable Substrates to the Mitochondria in ME/CFS Lymphoblasts

**DOI:** 10.3390/ijms22042046

**Published:** 2021-02-19

**Authors:** Daniel Missailidis, Oana Sanislav, Claire Y. Allan, Paige K. Smith, Sarah J. Annesley, Paul R. Fisher

**Affiliations:** 1 Department of Physiology, Anatomy and Microbiology, School of Life Sciences, La Trobe University, Melbourne, VIC 3086, Australia; D.Missailidis@latrobe.edu.au (D.M.); O.Sanislav@latrobe.edu.au (O.S.); Claire.Allan@latrobe.edu.au (C.Y.A.); S.Annesley@latrobe.edu.au (S.J.A.); 2 Monash Health, Melbourne, VIC 3186, Australia; paigeksmith81@gmail.com

**Keywords:** Myalgic Encephalomyelitis, ME/CFS, mitochondria, metabolism, transcriptomics, proteomics, beta-oxidation, amino acid catabolism, glycolysis, TCA cycle

## Abstract

Although understanding of the biomedical basis of Myalgic Encephalomyelitis/Chronic Fatigue Syndrome (ME/CFS) is growing, the underlying pathological mechanisms remain uncertain. We recently reported a reduction in the proportion of basal oxygen consumption due to ATP synthesis by Complex V in ME/CFS patient-derived lymphoblast cell lines, suggesting mitochondrial respiratory inefficiency. This was accompanied by elevated respiratory capacity, elevated mammalian target of rapamycin complex 1 (mTORC1) signaling activity and elevated expression of enzymes involved in the TCA cycle, fatty acid β-oxidation and mitochondrial transport. These and other observations led us to hypothesise the dysregulation of pathways providing the mitochondria with oxidisable substrates. In our current study, we aimed to revisit this hypothesis by applying a combination of whole-cell transcriptomics, proteomics and energy stress signaling activity measures using subsets of up to 34 ME/CFS and 31 healthy control lymphoblast cell lines from our growing library. While levels of glycolytic enzymes were unchanged in accordance with our previous observations of unaltered glycolytic rates, the whole-cell proteomes of ME/CFS lymphoblasts contained elevated levels of enzymes involved in the TCA cycle (*p* = 1.03 × 10^−4^), the pentose phosphate pathway (*p* = 0.034, G6PD *p* = 5.5 × 10^−4^), mitochondrial fatty acid β-oxidation (*p* = 9.2 × 10^−3^), and degradation of amino acids including glutamine/glutamate (GLS *p* = 0.034, GLUD1 *p* = 0.048, GOT2 *p* = 0.026), branched-chain amino acids (BCKDHA *p* = 0.028, BCKDHB *p* = 0.031) and essential amino acids (FAH *p* = 0.036, GCDH *p* = 0.006). The activity of the major cellular energy stress sensor, AMPK, was elevated but the increase did not reach statistical significance. The results suggest that ME/CFS metabolism is dysregulated such that alternatives to glycolysis are more heavily utilised than in controls to provide the mitochondria with oxidisable substrates.

## 1. Introduction

### 1.1. Background

Myalgic Encephalomyelitis/Chronic Fatigue Syndrome (ME/CFS) is a chronic disease characterized by debilitating fatigue and a worsening of symptoms following exertion referred to as post-exertional malaise (PEM). This can include pain, cognitive difficulty, flu-like symptoms and myriad other symptoms whose severity is disproportionate to the exertion-inducing activity. These properties of ME/CFS can significantly impair quality of life to the point of bed-bound disability in severe cases, rivalling the impact of other similarly devastating chronic diseases such as multiple sclerosis [1]. Much evidence for the biological basis of ME/CFS has been presented, but no underlying mechanism of disease has yet been identified. Insufficient cellular energy supply has been suspected, and in line with this, we previously reported inefficient ATP synthesis by Complex V in ME/CFS lymphoblasts [2].

### 1.2. Provisioning the Mitochondria

**Figure 1:**
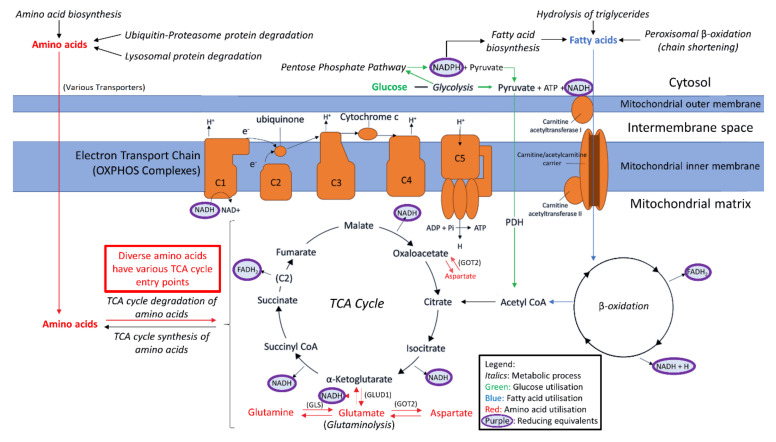
Simplified depiction of oxidisable substrate provision and usage by the mitochondria. The generalised “flow” of substrate molecules derived from glucose, fatty acids or amino acids is represented by arrows colour-coded green, blue and red, respectively. Reducing equivalents are denoted by purple. Processes are italicised. Glucose may be catabolised by glycolysis in order to provision the mitochondria with pyruvate, converted by pyruvate dehydrogenase (PDH) to acetyl CoA for entry into the TCA cycle. Fatty acid β-oxidation also provides acetyl CoA for the TCA cycle, by the catabolism of lipids rather than carbohydrates. The means by which amino acids may be similarly utilised are diverse and are described as appropriate throughout the text. However, we have highlighted glutamine usage in this figure due to its importance. Glutamine may be converted to glutamate by glutaminase. Glutamate may be converted to α-KG by glutamate dehydrogenase (GLUD1) for entry into the TCA cycle, or to aspartate by mitochondrial aspartate aminotransferase (GOT2) which is utilised in cellular redox balancing and TCA cycle anaplerosis, thereby providing both reducing equivalents for OXPHOS and intermediates for the TCA cycle. Reducing equivalents resultant from these myriad processes can deposit electrons into the electron transport chain to facilitate generation of the proton-motive force which drives ATP synthesis.

OXPHOS is the primary source of cellular ATP. This process is driven by the flow of electrons through Complexes I–IV, which mediates the pumping of protons out into the mitochondrial intermembrane space. This generates the electrochemical gradient utilised by Complex V to phosphorylate ADP to ATP. The electrons necessary for this process are deposited into the electron transport chain by the reducing equivalents NADH and FADH_2_. The provision of these electron donors to the OXPHOS complexes is therefore critical for ATP synthesis by aerobic respiration. The principle mitochondrial source of reduced NADH and FADH_2_ is the TCA cycle, which is supplied with metabolic intermediates at multiple entry points by a variety of nutrient metabolism pathways. The TCA cycle is thus a mitochondrial junction point for the participation of diverse fuel sources in respiration, including carbohydrates, fatty acids and amino acids (Figure 1).

In accordance with suspicions of insufficient cellular energy supply, the study of metabolism and pathways supplying the mitochondria with substrate in ME/CFS has increased in the last decade. Through the use of techniques such as nuclear magnetic resonance (NMR) or mass spectroscopy, quantitative snapshots of the metabolites present within a sample (commonly blood or urine) can be obtained. The relative levels of metabolites in patients versus controls can be subsequently used to infer which metabolic pathways may be upregulated, downregulated or bypassed. Metabolomic studies have been adopted by an increasing number of research groups in the ME/CFS field. Much of this work has discussed potential dysregulation of glycolysis in ME/CFS of glycolysis which mediates the multi-step conversion of glucose to pyruvate, which can be converted by pyruvate dehydrogenase (PDH) to acetyl CoA, a major TCA cycle substrate.

Armstrong et al. utilised ^1^H-NMR to assess the levels of metabolites in the serum and urine of ME/CFS patients compared to healthy controls [3,4]. Their results suggest an inhibition of glycolysis, which is consistent with a report by others using ME/CFS plasma, suggesting that utilisation of glycolytic pyruvate by the TCA cycle is reduced [5]. However, others using serum have proposed that impaired provision of glucose-derived acetyl CoA towards the TCA cycle is instead caused by an impairment of PDH function downstream of glycolysis, rather than an impairment of glycolysis itself [6]. Other studies have instead employed Seahorse respirometry to investigate real-time parameters of respiration and glycolysis in live cells from ME/CFS patients and compared to healthy controls. While the rate of glycolysis was found to be reduced in ME/CFS CD4^+^ and CD8^+^ T cells [7], we found no difference in glycolytic rate in ME/CFS lymphoblasts [2], nor did Tomas et al. in ME/CFS PBMCs or skeletal muscle cells [8,9]. Overall, the role of glycolysis in ME/CFS is unclear and would benefit from continued investigation.

In view of the inconsistent evidence for a specific glycolytic defect, the consideration of other processes involved in carbohydrate utilisation is also warranted. Others have reported reductions in the levels of 5/7 subgroups of metabolites involved in carbohydrate metabolism (including the disaccharide sucrose) in ME/CFS plasma samples versus those of healthy controls [10]. A reduction in the plasma levels of disaccharides in the energy-deficient context of ME/CFS could reflect broadly increased carbohydrate catabolism to satisfy elevated cellular glucose usage. If the rate of glycolysis itself was unaffected or impaired, glucose could instead be depleted by increased usage of the pentose phosphate pathway (PPP), which branches from glycolysis by the irreversible dehydrogenation of glucose-6-phosphate and involves the ATP-neutral synthesis of products crucial in cellular redox balancing and biosynthetic pathways [11]. Importantly, PPP products such as pyruvate can also be utilised to generate ATP, providing oxidisable substrates to the mitochondria. Indeed, the same authors who observed reduced plasma disaccharides in ME/CFS have previously suggested that the PPP may be dysregulated in ME/CFS [12]. This pathway should therefore also be examined more closely.

### 1.3. Alternative Sources of Oxidisable Substrates Than Carbohydrates

Amino acids may be metabolised to feed into the TCA cycle as sources of oxidisable substrate for respiration, or to participate in the replenishment of other metabolic intermediates. Glutamate is the metabolic product of multiple amino acids, prominent among which is glutamine [13,14]. The conversion of glutamine to glutamate by glutaminase (GLS) [14] and subsequent conversion to the TCA cycle intermediate α-ketoglutarate (α-KG) by glutamate dehydrogenase (GLUD1) is an important mechanism through which the TCA cycle can utilise amino acids to assist with driving mitochondrial energy production [15]. This reaction simultaneously reduces NAD^+^ to NADH, and so doubles as another direct means of replenishing reducing equivalents for OXPHOS. The functions of GLS and GLUD1 in tandem are therefore important for both direct mitochondrial NADH replenishment and as a major route of amino acid entry into the TCA cycle. This mechanism is regulated according to cellular energy demand, with GLUD1 activity mainly controlled allosterically-negatively by GTP and positively by ADP [16].

Cells may also utilise glutamate to both replenish reducing equivalents inside the mitochondria and assist in TCA cycle intermediate replenishment through participation in the malate-aspartate shuttle (MAS) [15,17]. Here, glutamate and oxaloacetate are converted to aspartate and α-KG by mitochondrial aspartate aminotransferase (GOT2). Aspartate is then transported out of the mitochondria and participates in the remainder of the cycle, which regenerates (a) cytosolic NAD^+^ from NADH, to be again reduced in catabolic non-mitochondrial processes such as glycolysis or peroxisomal β-oxidation and (b) mitochondrial NADH for OXPHOS through malate dehydrogenase in the TCA cycle. Glutamate therefore acts not only as a direct source of amino acid-derived NADH and TCA cycle substrate through the earlier described GLUD1 route, but also does so by provisioning the GOT/MAS route.

The aforementioned studies using serum by Fluge et al. and Armstrong et al. also suggested that catabolism of amino acids to feed the TCA cycle is more heavily utilised in ME/CFS patients [4,6]. However, the specifics differ. Armstrong et al. propose elevated glutamate usage by the mitochondria, specifically via the deamination of glutamate to aspartate as indicated by reduced glutamate and elevated aspartate levels (GOT2 route). By contrast, Fluge et al. observed reductions in the levels of both glutamine and glutamate but also in the levels of aspartate, which may instead suggest increased glutamate degradation through the GLUD1 route, rather than through GOT2 as suggested by Armstrong et al. However, perhaps in contradiction to this, Fluge et al. also reported elevated sirtuin 4 (*SIRT4*) mRNA expression in ME/CFS PBMCs and SIRT4 is known to suppress GLUD1 activity [18]. In spite of some inconsistent details, these studies highlight dysregulated amino acid metabolism as an important area of exploration which should be pursued further in ME/CFS, especially in a metabolically active cellular context.

The potential for abnormal utilisation of fatty acid β-oxidation has arisen clearly in our own work wherein we observed inefficient ATP synthesis by Complex V accompanied by a compensatory elevation of respiratory capacity and elevated expression of mitochondrial transporters, TCA cycle and fatty acid β-oxidation enzymes in ME/CFS lymphoblasts [2]. Fatty acid β-oxidation entails the breakdown of fatty acids to acetyl CoA for the TCA cycle while also replenishing reducing equivalents. While discussed in multiple metabolomic studies, lipid metabolism in general in ME/CFS research is also a point of uncertainty which requires re-examination. Naviaux et al. first reported that ceramide levels were decreased in ME/CFS patients, while Nagy-Szakal et al. subsequently did not observe a consistent decrease, and most recently Germain et al. reported an increase in ceramide levels [10,19,20]. Another discrepancy is that the reduced FAD levels reported by Naviaux et al. and reduced carnitines reported by Nagy-Szakal et al. are interpreted as likely to hinder fatty acid β-oxidation, while increased levels of the compound hexanoylglutamine reported by Germain et al. are suggested to instead indicate an upregulation of fatty acid β-oxidation in ME/CFS patients [10,19,20]. Germain et al. reported differences between two of their own studies, attributing these to differences in sample collection and handling [10,21]. This highlights the value of building and revisiting disease models in stably proliferative cell culture systems which are less affected by these sample collection issues and more readily reproducible.

Fatty acid β-oxidation is stimulated by AMP-activated protein kinase (AMPK) activity, one of the master regulators of energy metabolism in the cell [22]. Abnormal elevation of AMPK activity could explain the reported elevation of short-chain fatty acid levels in ME/CFS patients [23]. Elevated levels of phosphorylated (activated) AMPK were observed by Mensah et al. in particular subpopulations of B cells whose frequency in the B cell population was elevated in ME/CFS samples [24]. However, others have reported that the AMPK activation state was not significantly different between cultured muscle cells from CFS patients (Fukuda criteria) and healthy controls [25]. Additional study is therefore warranted to clarify the role of AMPK in ME/CFS.

### 1.4. Investigating Fuel Source Preference in ME/CFS Lymphoblasts

Amongst our previous findings of inefficient ATP synthesis, elevated respiratory capacity, non-mitochondrial catabolism, and increased expression of mitochondrial solute carriers, TCA cycle and β-oxidation enzymes in ME/CFS lymphoblasts, it became clear that the utilisation of other pathways providing substrates to the mitochondria were also likely to be dysregulated in these cells [2]. We subsequently embarked on combined transcriptomics and proteomics with the aims of identifying pathways which are dysregulated in ME/CFS lymphoblasts with greater clarity across both levels of regulation, and to verify the conclusions of our preliminary proteomics work [2] in a larger sample. We also assayed AMPK activity in ME/CFS lymphoblasts. Our observations herein suggest elevated usage of the PPP, fatty acid β-oxidation, and of the degradative mitochondrial pathways for specific amino acids. Together, these results seem to reflect a shift towards fatty acid and amino acid catabolism as the preferred sources of oxidisable substrate for the mitochondria in ME/CFS lymphoblasts.

## 2. Results

### 2.1. Global Changes in ME/CFS Lymphoblast Transcriptomes and Proteomes

**Figure 2:**
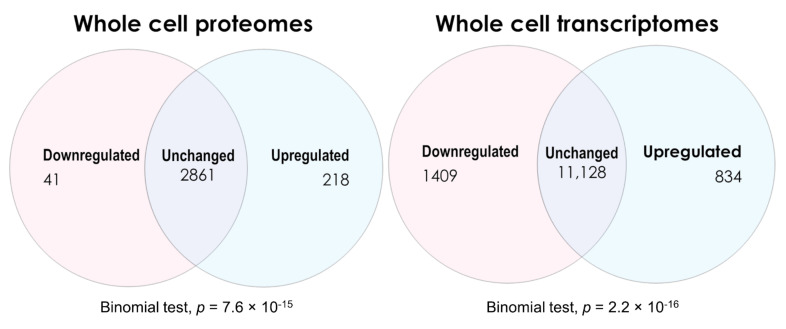
Venn diagrams depicting the numbers of differentially expressed gene products in Myalgic Encephalomyelitis/Chronic Fatigue Syndrome lymphoblasts within the whole-cell proteomics and transcriptomics experiments. Two-sided binomial tests were undertaken with H_o_ set to *p =* 0.5 to assess whether the differentially expressed fractions significantly departed from proportions expected by chance. Resulting significance probabilities (*p*) are indicated.

Up- and downregulated lists of genes and proteins from both the proteomics and transcriptomics experiments were determined by applying the Benjamini–Hochberg step-up correction for multiple comparisons (with *q* < 0.05) to the significance probabilities from *t* tests in the proteomics or F tests in the transcriptomics [26]. The numbers of differentially expressed vs. unchanged gene products detected in ME/CFS lymphoblasts across both types of experiment are shown in Figure 2. Comparing the whole-cell proteomes of ME/CFS and healthy control lymphoblasts revealed 218 upregulated, 41 downregulated, and 2861 proteins whose levels were unchanged. The expression of significantly more proteins was upregulated than downregulated (binomial test). Conversely, the transcriptomics revealed 843 transcripts upregulated, 1409 downregulated, and 11,128 unchanged—significantly more downregulated than upregulated (binomial test, null hypothesis—equal up- and downregulated proportions).

To obtain a broad perspective of pathway-level changes to guide subsequent analysis, the PANTHER over-representation tool [27,28,29] was employed to analyse outcomes from both the whole-cell transcriptomics and proteomics experiments. The entire list of detected genes or proteins from the respective experiment type was used as the “reference” list for comparison by PANTHER against the “input” lists of differentially expressed genes and proteins (*q* < 0.05). This produced a readout of pathways which are over-represented in the differentially expressed fractions of both datasets. The over-representation analysis applied a binomial test of the hypothesis that more genes or proteins in a given pathway are present in the respective “input” list than would be expected by chance, using their occurrence in the “reference” list as the expected proportion. The global pathway-level analysis has been included as Supplementary Materials and is summarised briefly throughout this section. Since the focus of this study was to investigate the metabolic provision of oxidisable substrates to the mitochondria, Table 1 highlights the PANTHER analysis of these particular metabolic and mitochondrial pathways of interest—carbohydrate metabolism, TCA cycle and respiration, other mitochondrial pathways, lipid metabolism, β-oxidation, amino acid metabolism, protein degradation, substrate transport and metabolism more broadly. The relevant pathways were subsequently revisited in-depth (see later).

#### 2.1.1. Transcriptomes

With additional correction for multiple comparisons (number of pathways) by a false discovery rate (FDR) cutoff of 0.05, 123 pathways were still statistically over-represented in the genes downregulated in ME/CFS lymphoblast transcriptomes (Table S1), while no pathways were over-represented in the upregulated genes. Contrasting with our prior observations of elevated protein-level expression in various pathways related to mitochondrial ATP synthesis in ME/CFS [2], these results showed that pathway expression at the transcript level, including those involved in mitochondrial respiration (Table 1, Table S1), is more broadly reduced in ME/CFS lymphoblasts.

Nevertheless, 834 individual transcripts were significantly upregulated (*q* < 0.05). We considered the possibility that FDR correction of the PANTHER pathway analysis of the upregulated transcript list was overly conservative. To identify which pathways might indeed show evidence of upregulation, we repeated the analysis with FDR correction excluded in the PANTHER analysis to identify candidate pathways. Of the resultant 51 pathways over-represented in this upregulated fraction (binomial test, *p* value < 0.05), most pertain to innate immune system activation or the import and intracellular transport of small molecules (Table S2). The latter could indicate homeostatic upregulation of the uptake of vitamins, sugars, amino acids or other small molecules important for sustaining cellular metabolism from the surrounding medium.

In view of a prior report of elevated *SIRT4* mRNA expression in ME/CFS PBMCs [6], we examined whether expression of any of the sirtuins was altered. In ME/CFS lymphoblasts, the expression levels of *SIRT4* as well as *SIRT1*, *SIRT5* and *SIRT7* were not significantly changed, while *SIRT2* was significantly upregulated and *SIRT3* and *SIRT6* were downregulated (Table S3). The sirtuins were not detected in the proteomics analysis, so it is unclear whether the levels of any sirtuin proteins are altered in ME/CFS lymphoblasts. It has also been reported that compared to controls, ME/CFS PBMCs exhibit different proportions of immune cell subtypes as detected by flow cytometry of CD cell surface markers [30,31]. We therefore also examined our data for differential expression of CD cell-surface markers and found that *CD19*, *CD47*, *CD52* and *CD79A* were significantly downregulated while only *CD164* was significantly upregulated in ME/CFS lymphoblasts (Table S3). Although all of these are expressed in activated B cells, none are markers for specific subsets of B cells [32], the immune cell type that is specifically infected by EBV during immortalisation. None of them were differentially expressed in the whole-cell proteomes (see below).

#### 2.1.2. Proteomes

Similar analysis of differential pathway expression in the whole-cell proteomes showed highly distinct results from the transcriptomes and confirmed the conclusions of our previously published preliminary proteomics work [2]. While the lists of up- and downregulated proteins were also conservatively selected using the *q* < 0.05 method, additional FDR correction of the pathway over-representation tests was excluded in the proteomic pathway analysis. This was performed as the additional FDR correction at the pathway level was found to be overly conservative and obscured true positives which were separately confirmed by closer analysis of individual pathways, and in the case of OXPHOS proteins also by our prior multi-pronged tests of activity and expression [2]. These true-positive pathways satisfied a *p* < 0.05 threshold for the binomial test of over-representation when the pathway FDR was not used. This analysis identified 77 pathways over-represented in the significantly upregulated fraction of proteins (Table S4), while 13 pathways were over-represented in the downregulated fraction (Table S5).

Of the downregulated fraction, most of the 13 pathways were represented by very few detected proteins or proteins that were significantly downregulated or were pathway hits irrelevant in the context of lymphoid cells (such as meiosis). The most significantly affected pathway with both tissue-specific relevance and a greater number of downregulated proteins was the activation of protein kinase Ns (PKNs) by RHO GTPases (Reactome pathway R-HSA-5625740). This could suggest reduced activation of PKNs in ME/CFS lymphoblasts. The various PKNs, while involved in signal transduction related to many processes such as cell migration and cytoskeleton assembly, also play roles in transcriptional activation which have been most clearly observed in cardiac tissue [33]. If fulfilling similar roles in lymphoid cells, reduced PKN activation could be a contributor to the largely reduced transcript-level expression apparent in ME/CFS lymphoblasts.

By contrast, the 77 pathways over-represented in the upregulated protein fraction possessed proteins which were altered significantly in much higher numbers than pathways in the downregulated fraction and were more informative in their biological context (Table S4). Most strikingly, 9/20 of the most significantly upregulated pathways pertained directly to fatty acid β-oxidation (Table 1, Table S4). This strongly suggests an upregulation of fatty acid β-oxidation in ME/CFS lymphoblasts, which is consistent with our preliminary work [2].

Another seven out of twenty of the most significantly upregulated pathways pertained to activation of both the innate and adaptive immune responses (Table S4) as was also evident in the transcriptomes, together suggesting elevated immune activation in ME/CFS lymphoblasts. Of several CD antigens detected in the proteomics, most trended upwards although only the expression of CD226, CD48 and CD70 were individually altered significantly (upregulated) in ME/CFS lymphoblasts (Table S6). None of these were differentially expressed in transcriptomes of ME/CFS lymphoblasts, none are markers of specific B cell subsets [32] but are expressed on proliferating, activated B cells. CD70 expression is a marker of highly activated lymphocytes [34], and its higher expression in ME/CFS lymphoblasts is thus in keeping with the (presumably compensatory) general hyperactivation of metabolism in these cells.

Additionally prominent among the list of significantly upregulated pathways were mitochondrial biogenesis (R-HSA-1592230), the TCA cycle and respiratory electron transport (R-HSA-1428517) and other mitochondrial proteins (Table 1, Table S4). This was as expected given our previous observations of elevated respiratory capacity and broadly elevated mitochondrial protein expression, including in the TCA cycle and OXPHOS complex subunits specifically [2]. The PPP was also found to be over-represented in this analysis, in line with previous proposals of a metabolic shift by ourselves and others [2,12] (Table 1, Table S4). At least five of the over-represented pathways were reflective of abnormal amino acid metabolism or degradation, which implied dysregulated amino acid usage by ME/CFS lymphoblasts as we previously hypothesised [2] (Table 1, Table S4).

We previously proposed that ATP synthesis by Complex V was inefficient in ME/CFS lymphoblasts, and that this was accompanied by compensatory upregulation of mitochondrial protein expression and the dysregulation of substrate-providing pathways. In the absence of a glycolytic rate abnormality and in the presence of elevated fatty acid β-oxidation enzyme expression, we proposed that ME/CFS lymphoblasts may increasingly rely upon fatty acids or other alternatives to glycolysis to supply the upregulated TCA cycle and respiratory electron transport complexes with substrate at faster rates. The foregoing exploratory analysis at the global proteome level using our new, larger dataset is not only consistent with these conclusions, but also more strongly suggests the upregulation of other alternatives such as the PPP or amino acid catabolism in order to provide ME/CFS lymphoblast mitochondria with oxidisable substrate. The broad trends apparent in the whole-cell transcriptomics also indicate that elevated levels of the proteins in such pathways could occur as a result of upregulation specifically at the translational level. We used the foregoing observations as a basis to guide the subsequent, closer analysis of specific pathways of interest to understand how provision of mitochondrial substrates could be dysregulated in ME/CFS lymphoblasts in more detail.

### 2.2. Confirming Previous Protein-Level Expression Results and Examining Transcript Levels in Key Mitochondrial Pathways

We began the more targeted analysis of individual pathways by exploring functional groups which we previously found by proteomics or western blotting to be upregulated and most closely related to the other mitochondrial abnormalities we observed in ME/CFS lymphoblasts [2]. These groups were the five OXPHOS complexes, the TCA cycle, and mitochondrial transporters. Analysis of our enlarged proteomics dataset confirmed the upregulation of all five OXPHOS Complexes, of TCA cycle enzymes taken as a whole, and of mitochondrial solute carrier family (SLC25) members, while subunits of the mitochondrial protein import complexes translocase of the inner mitochondrial membrane (TIMM), translocase of the outer mitochondrial membrane (TOMM) and sorting and assembly machinery (SAMM) were not upregulated at the protein level (Table 2). Together, this reaffirms the compensatory upregulation of respiratory complexes and TCA cycle enzymes as we previously proposed, and that ME/CFS lymphoblast mitochondria upregulate their import of small molecules, including oxidisable substrates, by SLC25 transporters. The upregulation of SLC25 transporters but not the TIMM, TOMM and SAMM protein import machinery is consistent with our previous observation that the mitochondrial “mass” is unchanged in ME/CFS lymphoblasts, and with the hypothesis that their use of oxidisable substrates provided by pathways other than glycolysis is elevated. It also suggests that the elevated proton leak (use of the mitochondrial proton gradient for purposes other than ATP synthesis) and depletion of the mitochondrial membrane potential [2] may be more largely due to the upregulated import of small molecules than of proteins.

When expression of these pathways was assessed at the transcript level (Table 3), Complex II expression was elevated, the expression of SLC25 and TCA cycle enzyme expression was unchanged, while Complexes I, III, IV, V and the mitochondrial protein import complexes were actually downregulated. Except for Complex II, we conclude that the upregulation of mitochondrial OXPHOS must result from processes at the posttranscriptional level. Clearly Complex II expression is regulated differently from the other respiratory complexes, a difference that makes sense given that expression of Complexes I, III, IV and V is known to be coordinately regulated, while control of Complex II expression is more closely coupled to that of the other TCA cycle enzymes [35]. The most likely mechanism for posttranscriptional upregulation of most central OXPHOS proteins is that their rates of translation are elevated by the increased mTORC1 activity we previously reported in ME/CFS lymphoblasts [2]. mTORC1 can stimulate the expression of mitochondrial proteins both 1) indirectly at the transcriptional level by upregulating translation of the transcription factors PGC1α and TFAM [36] and 2) directly at the translational level by activating the translational activator S6 kinase (S6K) and inhibiting the translational repressor 4E-BP1, both by phosphorylation [37,38].

We previously verified the upregulation of OXPHOS subunits at the protein level by Western blotting [2]. We carried out further Western blots and qRT-PCR experiments to respectively verify the new proteomic and transcriptomic datasets. Using Western blotting, both ACO2 and SDHA showed the same absence of altered expression as in the proteomic data, while elevated levels of MDH1 in the proteomes were also confirmed (Supplementary Figures S1A and S2). At the transcript level, the directional trends of *SDHB, GLS, NDUFB1* and *NDUFB10* normalised to the histone gene *HIST1H1C* were confirmed in all four cases between qRT-PCR experiments and the transcriptomic dataset (Figure S1B).

### 2.3. Expression of Enzymes Involved in Carbohydrate Catabolism by Glycolysis and the Pentose Phosphate Pathway (PPP)

**Figure 3:**
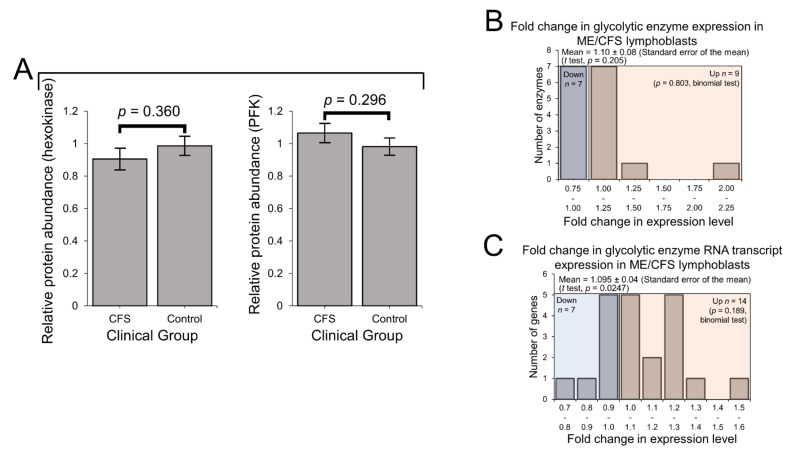
Protein-level expression of glycolytic enzymes is unchanged in Myalgic Encephalomyelitis/Chronic Fatigue Syndrome (ME/CFS) lymphoblasts. Error bars represent the standard errors of the mean. RNA sequencing transcriptomics experiment: ME/CFS *n* = 23, control *n* = 17. Each cell line was sampled once. Mass spectrometry proteomics experiment: ME/CFS *n* = 34, control *n* = 31. Each cell line was sampled once, or twice for a subset of healthy controls arbitrarily selected to act as an internal control between experiments in the proteomics work. (**A**) The expression level of hexokinase and phosphofructokinase is unchanged in whole-cell mass spectrometry proteomics experiments (independent *t test*). Relative hexokinase (HK) and phosphofructokinase (PFK) abundance was calculated by averaging the mean fold change in the ME/CFS group for each isoenzyme/subunit of the respective enzyme (none of which were statistically significant on their own, threshold *p* < 0.05). (**B**) A total of 16 glycolytic enzymes were detected within the whole-cell proteomes of lymphoblasts from ME/CFS and control lymphoblasts. Fold change refers to the mean abundance of a given protein in the CFS group divided by the mean abundance in the control group. There was no significant difference in the differentially expressed proportions of detected glycolytic enzymes (binomial test with H_o_ set to 0.5) or the magnitude of expression (single-sample *t* test with H_o_ *m* = 1) between ME/CFS and controls. (**C**) A total of 21 RNA transcripts encoding glycolytic enzymes were detected by RNA sequencing within the whole-cell transcriptomes of ME/CFS and control lymphoblasts. Mean fold change was calculated for the ME/CFS group versus the control average for each transcript. The proportion of detected transcripts that were upregulated (binomial test with H_o_ set to 0.5) was not significant while the average extent of the upregulation (single-sample *t* test with H_o_ *m* = 1) was statistically significant.

We previously reported that the rate of glycolysis as measured by respirometry was unchanged in ME/CFS lymphoblasts, despite elevated non-mitochondrial catabolic rates and mitochondrial solute carrier expression [2]. This suggested that other alternatives were being increasingly utilised to provision the mitochondria, compared to glycolysis. Here, using the lymphoblast proteomics and transcriptomics, we investigated the expression of glycolytic enzymes both at the whole-pathway level and with respect to individual rate-determining enzymes. In keeping with the unchanged rate of glycolysis [2], expression of the two key rate-controlling enzymes of glycolysis—phosphofructokinase and hexokinase—was unchanged from that of controls (Figure 3A). Indeed, the levels of all detected glycolytic enzymes (including subunits and isoenzymes: 16 proteins) were also found to be unchanged as a whole in the ME/CFS lymphoblast proteomes (*p* > 0.05, *t* test and binomial test) (Figure 3B). This contrasts with slightly elevated levels of mRNAs encoding these enzymes which we observed in the whole-cell transcriptomes (Figure 3C). Here, 14/21 detected transcripts favoured upregulation with mean levels 10 ± 4% higher than controls (*t* test, *p* = 0.0247), 6 of which encoded isoenzymes or subunits of phosphofructokinase and hexokinase. It has been shown by others that the accumulation of untranslated mRNAs encoding glycolytic enzymes can occur when lymphoid cells are in a metabolic state favouring fatty acid β-oxidation rather than glycolysis [39]. If ME/CFS lymphoblasts do indeed favour fatty acid β-oxidation instead of glycolysis, this would be consistent with the pattern of expression observed here.

**Figure 4:**
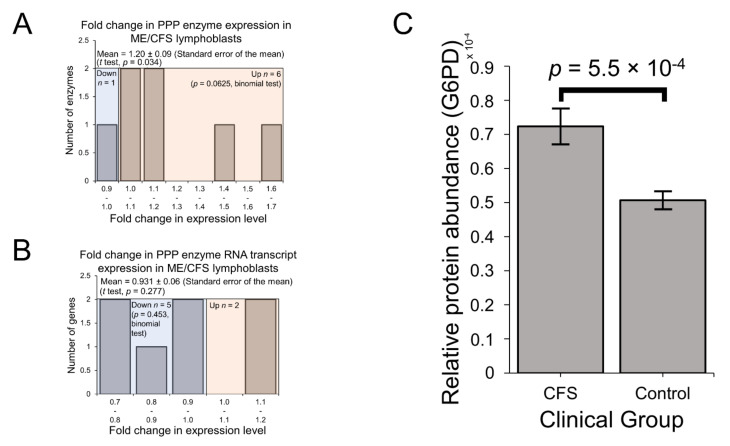
Expression of the pentose phosphate pathway (PPP) is upregulated at the protein level in Myalgic Encephalomyelitis (ME/CFS) lymphoblasts. Error bars represent the standard error of the mean. Mass spectrometry proteomics experiment: ME/CFS *n* = 34, control *n* = 31. Each cell line was sampled once, or twice for a subset of healthy controls arbitrarily selected to act an internal control between experiments. (**A**) A total of 7 PPP enzymes were detected within the whole-cell proteomes of lymphoblasts from ME/CFS and control lymphoblasts. Fold change refers to the mean abundance of a given protein in the CFS group divided by the mean abundance in the control group. There was no significant difference in the proportion of upregulated PPP enzymes (binomial test with H_o_ set to 0.5), but the magnitude of upregulation was significantly elevated in ME/CFS lymphoblasts (single-sample *t* test with H_o_ m ≤1 and H_1_ m>1, *p* = 0.034). (**B**) A total of 7 RNA transcripts encoding PPP enzymes were detected by RNA sequencing within the whole-cell transcriptomes of ME/CFS and control lymphoblasts. Mean fold change was calculated for the ME/CFS group versus the control average for each transcript. The proportions of reduced or elevated transcripts were not significantly different (binomial test with H_o_ set to 0.5) nor was the average magnitude of expression (single-sample *t* test with H_o_ *m* = 1). (**C**) The expression level of G6PD is significantly elevated (*t* test, *p* = 5.5 × 10^−4^) in whole-cell mass spectrometry proteomics experiments (independent *t test*). Relative abundance was obtained from Intensity-Based Absolute Quantitation values normalised to the control average within the respective individual experiments.

Glucose tracer experiments by others have shown that when B cells switch their metabolism in favour of fatty acid β-oxidation instead of towards glycolysis, glucose is utilised at the same overall rates, but by the PPP instead [40,41]. In ME/CFS research, reports of reduced glucose [4] and disaccharides [10] in patient blood could reflect elevated utilisation of the PPP if the rate of glycolysis is indeed unchanged in patient cells as we have observed. In any case, compensatory provision of additional pyruvate is one means by which the PPP may be utilised to support respiration as a source of oxidisable substrate in ME/CFS lymphoblast mitochondria whose mitochondrial ATP synthesis is inefficient [2]. PPP enzymes were also significantly over-represented among the upregulated fraction of proteins across the entire proteomics experiment (Table S4). This was confirmed when we selected all PPP enzymes that were detected in the proteome and examined their expression levels in the lymphoblast proteomes and transcriptomes. With mean levels 20 ± 9% higher than controls (*t* test, *p* = 0.034) (Figure 4A), PPP enzymes were significantly upregulated in the proteomes of ME/CFS lymphoblasts, while their expression was not significantly altered at the transcriptional level (Figure 4B). Importantly, glucose-6-phosphate 1-dehydrogenase (G6PD), the enzyme catalysing the first and rate-limiting step of the oxidative arm of the PPP [42], was significantly elevated on its own by 43 ± 10% in the proteomes of ME/CFS lymphoblasts versus healthy controls (*p* = 5.5 × 10^−4^) (Figure 4C). This suggests that the PPP is indeed upregulated as an alternative means of glucose utilisation to glycolysis in ME/CFS lymphoblasts.

### 2.4. Enzymes Involved in Mitochondrial and Fatty Acid β-Oxidation Are Elevated in Their Expression

**Figure 5:**
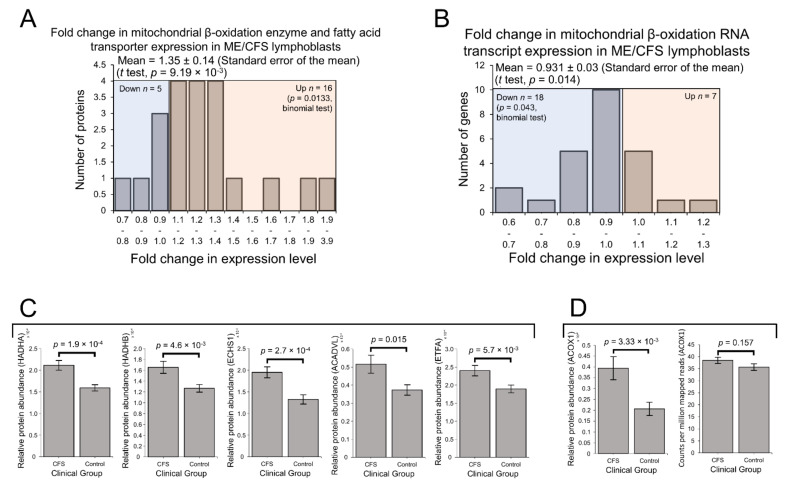
Expression of proteins involved in mitochondrial and peroxisomal fatty acid β-oxidation was elevated in Myalgic Encephalomyelitis/Chronic Fatigue Syndrome (ME/CFS) lymphoblasts. Error bars represent the standard error of the mean. RNA sequencing transcriptomics experiment: ME/CFS *n* = 23, control *n* = 17. Each cell line was sampled once. Mass spectrometry proteomics experiment: ME/CFS *n* = 34, control *n* = 31. Each cell line was sampled once, or twice for a subset of healthy controls arbitrarily selected to act an internal control between experiments in the proteomics work. (**A**) A total of 21 proteins involved in mitochondrial fatty acid β-oxidation and transport were detected within the whole-cell proteomes of ME/CFS and control lymphoblasts. Fold change refers to the mean abundance of a given protein in the CFS group divided by the mean abundance in the control group. The proportion of detected proteins that were upregulated (binomial test with H_o_ set to 0.5 and H_1_ being that the upregulated proportion was greater) and the average extent of the upregulation (single-sample *t* test with H_o_ ≤ m1 and H_1_ m>1) were statistically significant. (**B**) A total of 25 RNA transcripts encoding proteins involved in mitochondrial fatty acid β-oxidation and transport were detected by RNA sequencing within the whole-cell transcriptomes of ME/CFS and control lymphoblasts. Fold change refers to the mean abundance of a given transcript in the CFS group divided by the mean abundance in the control group. The proportions of reduced or elevated transcripts were not significantly different (binomial test with H_o_ *p* = 0.5) nor was the average magnitude of expression (single-sample *t* test with H_o_ *m* = 1). (**C**) The expression levels of both subunits of the mitochondrial trifunctional enzyme complex Hydroxyacyl-CoA dehydrogenase/3-keotacyl-CoA thiolase (HADHA and HADHB), very long-chain specific acyl-CoA dehydrogenase (ACADVL), enoyl-CoA hydratase (ECHS1) and electron transfer flavoprotein subunit alpha (ETFA (are significantly elevated in whole-cell mass spectrometry proteomics experiments (independent *t test*). Relative abundance was obtained from Intensity-Based Absolute Quantitation values normalised to the control average within the respective individual experiments. (**D**) The expression of Acyl-CoA oxidase 1 (ACOX1) was significantly elevated in whole-cell mass spectrometry proteomics experiments (independent *t test*). Relative abundance was obtained from Intensity-Based Absolute Quantitation values normalised to the control average within the respective individual experiments. *ACOX1* expression was not altered at the transcriptional level as measured by whole-cell RNA sequencing transcriptomics. Counts per million mapped reads were calculated for each gene transcript.

Enzymes involved in mitochondrial fatty acid β-oxidation were upregulated in ME/CFS lymphoblasts in our preliminary proteomics experiment [2]. Preferential fatty acid β-oxidation is the canonical metabolic alternative to glycolysis and is consistent with the previously reported upregulation of the PPP in B cells [40,41], from which lymphoblasts are derived. Given also its prominence in the exploratory pathway over-representation analysis, we strongly anticipated the expression of enzymes and transporters involved in mitochondrial fatty acid β-oxidation would be elevated in this larger sample of ME/CFS lymphoblasts. Indeed, here we found expression of these proteins to be significantly elevated in the ME/CFS group (Figure 5A). A total of 16 of the detected 21 proteins were upregulated (binomial test, *p* = 0.0133), with mean levels 35 ± 14% higher than controls (*t* test, *p* = 9.19 × 10^−3^). In contrast with the elevated protein expression and in keeping with the broader transcriptional trends, the levels of mRNA transcripts encoding these enzymes was found to be slightly reduced (Figure 5B). A total of 18 of the detected 25 transcripts were downregulated (binomial test, *p* = 0.043) with mean levels 9 ± 3% lower than controls (*t* test, *p* = 0.014). This suggests upregulation specifically at the translational level, as was apparent with other groups of proteins such as OXPHOS Complexes I, III, IV and V.

To better understand the potential functional implications of these differences in mitochondrial β-oxidation enzyme expression, we investigated more closely the expression of these proteins on an individual basis. Among the detected mitochondrial β-oxidation proteins, we found that the expression of five specific enzymes was significantly altered at the protein level individually, and that each of these was upregulated in the ME/CFS lymphoblasts (Figure 5C). Both subunits of the mitochondrial trifunctional enzyme (HADHA and HADHB) were among these five significantly upregulated proteins. Hydroxyacyl-CoA dehydrogenase/3-keotacyl-CoA thiolase (HADH) is an enzyme complex which catalyses multiple reactions in mitochondrial β-oxidation, exhibits specificity for long-chain fatty acids and is involved in cardiolipin synthesis—cardiolipin being an important component of the inner mitochondrial membrane [43,44]. Short-chain enoyl-CoA hydratase (ECHS1) was also significantly upregulated, as well as the very long-chain-specific acyl-CoA dehydrogenase (ACADVL). These, together with upregulated HADH expression, demonstrate the upregulation of enzymes involved in catabolising fatty acids of diverse chain-lengths, implying their complete oxidation within the mitochondria and thus their ultimate contribution towards the electron transport chain. This conclusion is reinforced by the remaining member of the five significantly upregulated proteins being the alpha subunit of the electron transfer flavoprotein (ETFA), which accepts electrons from the mitochondrial dehydrogenases involved in β-oxidation passing them via electron transfer flavoprotein-ubiquinone oxidoreductase (ETF-QO) and ubiquinone to Complex III in the electron transport chain [45].

Mitochondrial β-oxidation itself does not readily act upon very long-chain fatty acids (VLCFA), which are first chain-shortened by peroxisomal β-oxidation to then be utilised in either mitochondrial β-oxidation or directly in the TCA cycle as acetyl CoA [46]. Both mitochondrial and peroxisomal fatty acid β-oxidation, pathways which operate in tandem, are together upregulated by AMPK activity and the nuclear transcription factor peroxisome proliferator-activated receptor-α (PPAR-α) [22,47,48,49]. If ME/CFS lymphoblasts do switch their oxidisable substrate preference in favour of mitochondrial fatty acid β-oxidation, one would therefore also expect peroxisomal β-oxidation to be upregulated alongside it due to their shared regulatory mechanisms and entwined function. To investigate this, we more closely assessed the expression of individual enzymes involved in peroxisomal β-oxidation.

Acyl-CoA oxidase 1 (ACOX1) is the enzyme which first initiates VLCFA β-oxidation inside the peroxisome and is the rate-controlling enzyme of this process [50]. We found that the expression of ACOX1 was significantly upregulated in the proteomes by 91 ± 26% and unchanged in the transcriptomes of ME/CFS lymphoblasts (Figure 5D). Of the other enzymes involved in peroxisomal β-oxidation, most were not detected in either experiment, or were detected at low levels in only a few samples. As a result of their relatively poor detection, differences between ME/CFS and controls could not be found for these enzymes. Nonetheless, the significant upregulation of the pathway-initiating and rate-controlling enzyme ACOX1, in addition to the functional and regulatory coupling of these pathways described earlier, suggests that peroxisomal β-oxidation is likely to be upregulated in tandem with mitochondrial β-oxidation in ME/CFS lymphoblasts.

As noted above, if the rates of fatty acid β-oxidation are elevated in ME/CFS lymphoblasts in accordance with the expression of these enzymes, this would be consistent with the accumulation of untranslated glycolytic mRNA transcripts. More importantly, our observations here support our previous proposal that upregulated fatty acid β-oxidation in ME/CFS cells provides acetyl CoA to the TCA cycle more rapidly, provisioning the upregulated respiratory complexes with reducing equivalents to accelerate respiration and compensate for inefficient ATP synthesis by Complex V [2].

**Figure 6:**
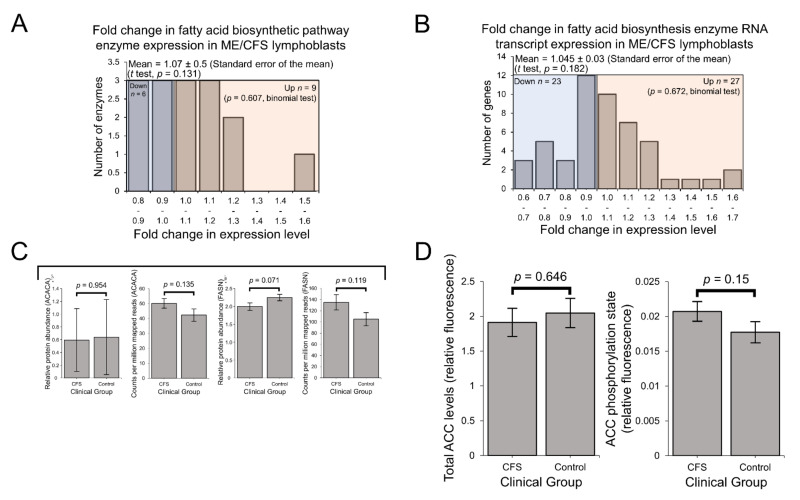
Expression of enzymes involved in mitochondrial fatty acid biosynthesis was elevated in the whole-cell transcriptomes but unchanged in the whole-cell proteomes of ME/CFS lymphoblasts. AMPK activity is not significantly elevated in ME/CFS lymphoblasts. Error bars represent the standard error of the mean. RNA sequencing transcriptomics experiment: ME/CFS *n* = 23, control *n* = 17. Each cell line was sampled once. Mass spectrometry proteomics experiment: ME/CFS *n* = 34, control *n* = 31. Each cell line was sampled once, or twice for a subset of healthy controls arbitrarily selected to act an internal control between experiments in the proteomics work. (**A**) A total of 15 proteins involved in fatty acid biosynthesis were detected within the whole-cell proteomes of ME/CFS and control lymphoblasts. Fold change refers to the mean abundance of a given protein in the CFS group divided by the mean abundance in the control group. The proportion of detected proteins that were differentially expressed (binomial test with H_o_ set to 0.5) and the average extent of any differences (single-sample *t* test with H_o_ *m* = 1) were not statistically significant. (**B**) A total of 51 RNA transcripts encoding proteins involved in fatty acid biosynthesis were detected by RNA sequencing within the whole-cell transcriptomes of ME/CFS and control lymphoblasts. Mean fold change was calculated for the ME/CFS group versus the control average for each transcript. The proportions of reduced or elevated transcripts were not significantly different (binomial test with H_o_ set to 0.5) nor was the average magnitude of expression (single-sample *t* test with H_o_ *m* = 1). (**C**) The expression of ACACA and FASN, two key enzymes involved in fatty acid biosynthesis, was not significantly altered in whole-cell proteomics or transcriptomics experiments (independent *t test*). Relative abundance was obtained from Intensity-Based Absolute Quantitation values normalised to the control average within the respective individual experiments. per million mapped reads were calculated for each gene transcript (**D**) AMPK activity is not significantly elevated in ME/CFS lymphoblasts. Total ACC levels were unaltered. AMPK activity was determined by measuring the ACC phosphorylation state normalised to total ACC levels in ME/CFS lymphoblasts (*n* = 28) and healthy controls (*n* = 24). Each cell line was measured in at least three independent experiments. Fluorescence is expressed in relative terms as each experiment is normalised to an internal control cell line.

Since fatty acid metabolism hinges on the controlled balance between biosynthesis and β-oxidation [22], it is also important to consider the pathways involved in fatty acid biosynthesis when drawing conclusions as to the favoured direction of fatty acid metabolism. Contrasting with the upregulation of β-oxidation enzymes, no significant differences were detected at either the protein level (Figure 6A) or the transcriptional level (Figure 6B) when assessing the expression of enzymes involved in fatty acid biosynthesis as a whole.

Particularly important among the involved enzymes are acetyl-CoA carboxylase 1 (ACC1), which catalyses the synthesis of malonyl-CoA—a key, rate-limiting substrate for fatty acid synthesis, and fatty acid synthase (FASN), which in turn catalyses the conversion of malonyl-CoA to palmitate [51,52]. Thus, these reactions control the rate of de novo fatty acid biosynthesis by the cell and their expression levels are important to investigate on an individual basis to assist with inferring functional consequences. In the whole-cell proteomes, the expression of ACC1 (ACACA) in ME/CFS lymphoblasts was unchanged compared to controls (albeit detected at low levels) and was also unchanged at the transcriptional level (Figure 6C). The unchanged expression of the ACC protein was later confirmed in a plate-based fluorescence assay (Figure 6D). Expression levels of FASN were also not significantly different at either the protein or transcript level (Figure 6C). Since fatty acid biosynthesis is selectively activated in competition with fatty acid β-oxidation [22,53] and mitochondrial fatty acid β-oxidation enzymes are upregulated in ME/CFS lymphoblasts, it is unsurprising that expression of these enzymes involved in fatty acid biosynthesis are not elevated. These observations together suggest that in ME/CFS lymphoblasts compared to controls, fatty acid metabolism is indeed operating in favour of β-oxidation rather than biosynthesis.

While mitochondrial enzymes, including those involved in fatty acid β-oxidation, are amongst those whose expression is upregulated by mTORC1 activity [36,54], their expression levels are not the only arbiters of the rates at which these pathways operate. The activity of AMPK is also important since it inactivates ACC by phosphorylation to inhibit fatty acid synthesis and promote fatty acid β-oxidation by preventing ACC from inhibiting fatty acid import into the mitochondria [22]. To investigate this, we measured AMPK activity in ME/CFS and control lymphoblasts by assaying the phosphorylation state of ACC. We found that the mean ACC phosphorylation state was elevated by ~17% in ME/CFS lymphoblasts, but this elevation did not reach statistical significance in our experiments (Figure 6D). This would suggest that the balance between fatty acid biosynthesis and β-oxidation is not significantly altered in ME/CFS cells. However, it remains possible that the elevation of ACC inactivation is real and sufficient to contribute to tipping the balance of fatty acid metabolism in favour of β-oxidation. This merits further investigation as it would be consistent with the elevated capacity of ME/CFS mitochondria for β-oxidation as shown by the elevated levels of the enzymes involved.

### 2.5. Expression of Enzymes Involved in the Mitochondrial Utilisation of Glutamine, BCAAs and Essential Amino Acids Is Elevated in ME/CFS Lymphoblasts

In ME/CFS, the increased utilisation of glutamine/glutamate through both the GLUD1 and GOT2 routes has been suggested through the outcomes of metabolomic studies [3,4,6]. If these processes are upregulated in ME/CFS, mitochondrial amino acid catabolism could be another means by which ME/CFS lymphoblasts compensate for their inefficiency of ATP synthesis. Indeed, the electron transfer flavoprotein (ETF), whose expression is elevated in ME/CFS lymphoblasts (Figure 5C), also accepts electrons derived from the oxidation of amino acids such as lysine and tryptophan to be donated to the electron transport chain through enzymes such as glutaryl-CoA dehydrogenase (GCDH) [55,56]. Furthermore, mTORC1, whose activity is elevated in ME/CFS lymphoblasts, is activated by glutamine catabolism [57,58] and has been shown to be essential for branched-chain amino acid (BCAA) catabolism in mice [59]. These factors together with the observed respiratory abnormalities and elevated TCA cycle enzyme expression strongly indicate the potential importance of energy-yielding amino acid catabolism in ME/CFS lymphoblasts. Since the PANTHER analysis of upregulated proteins in ME/CFS cells revealed a significant over-representation of pathways involved in amino acid metabolism (Table S4), we more closely assessed the expression of individual pathways and enzymes involved in these processes in the whole-cell proteomes and transcriptomes.

**Figure 7:**
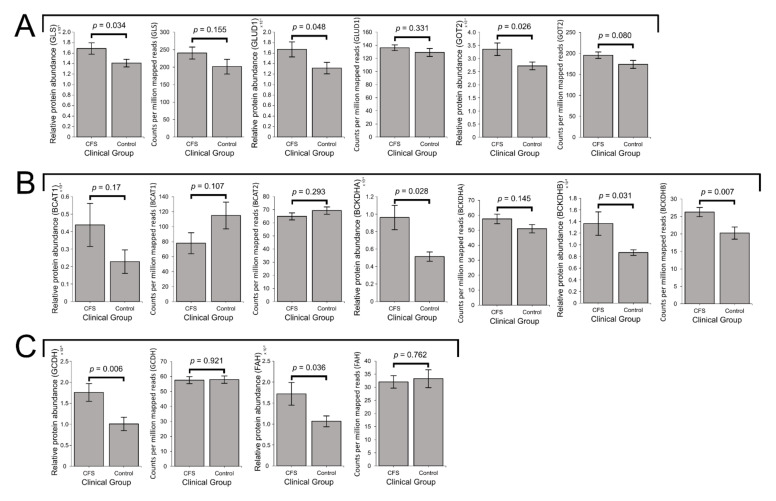
Expression of proteins involved in mitochondrial glutamine, BCAA, lysine, tryptophan and phenylalanine utilisation are elevated in ME/CFS lymphoblasts. Error bars represent the standard error of the mean. RNA sequencing transcriptomics experiment: ME/CFS *n* = 23, control *n* = 17. Each cell line was sampled once. Mass spectrometry proteomics experiment: ME/CFS *n* = 34, control *n* = 31. Each cell line was sampled once, or twice for a subset of healthy controls arbitrarily selected to act an internal control between experiments in the mass spectrometry proteomics work. (**A**) Expression of the three enzymes mediating mitochondrial utilisation of glutamate (GLS, GLUD1 and GOT2) were elevated in the whole-cell proteomes and proteomes of ME/CFS lymphoblasts and control lymphoblasts (*t* test, *p* < 0.05 in all three cases), while each trended upwards but were not significantly elevated at the transcript level. Relative protein abundance was obtained from Intensity-Based Absolute Quantitation values normalised to the control average within the respective individual experiments. Counts per million mapped reads were calculated for each gene transcript. (**B**) In ME/CFS lymphoblasts, the expression of BCAT1 is unchanged at the protein and transcript levels, while BCAT2 was unchanged transcriptionally and not detected at the protein level. The levels of BCKDH subunits BCKDHA and BCKDHB are both significantly elevated at the transcriptional and protein levels (*t* test, *p* < 0.05), with the exception of BCKDHA transcripts. Relative protein abundance was obtained from Intensity-Based Absolute Quantitation values normalised to the control average within the respective individual experiments. Counts per million mapped reads were calculated for each gene transcript (**C**) The expression levels of GCDH and FAH were unchanged at the transcriptional level but elevated at the protein level (*t* test, *p* < 0.05) in ME/CFS lymphoblasts. Relative protein abundance was obtained from Intensity-Based Absolute Quantitation values normalised to the control average within the respective individual proteomics experiments. Counts per million mapped reads were calculated for each gene transcript.

As detailed earlier, the key enzymes involved in glutamine/glutamate degradation are 1) GLS, responsible for metabolising glutamine to glutamate, 2) GLUD1, which converts glutamate to the TCA cycle substrate α-KG and replenishes NADH, and 3) GOT2, the mitochondrial enzyme simultaneously catalysing the conversion of glutamate to aspartate and oxaloacetate to α-KG (Figure 1). In ME/CFS lymphoblasts, the levels of each of these three enzymes were significantly elevated in the proteomes (*p* < 0.05 in all cases, fold increases ranging from 20 to 27%), while the levels of the transcripts encoding them also trended upwards but did not reach statistical significance in all three cases (Figure 7A). Together with the absence of changes in glycolysis, this observation may confirm previous proposals that in ME/CFS, relative to glycolysis, specific amino acids and their derivatives are more heavily utilised as a mitochondrial fuel source [4].

Glutamate can also be reversibly depleted or produced by the transamination activity of the branched-chain amino acid (BCAA) aminotransferases (BCATs). Like glutamate, BCAAs themselves may also be catabolised to provide the TCA cycle with substrate, the degradation of BCAAs being initiated by BCAT [60]. BCAT catalyses the reversible conversion (by transamination) of BCAAs to their respective branched-chain ketoacids, which are precursors of TCA cycle intermediates in BCAA degradation. When the BCAT-catalysed reaction runs in the direction favouring BCAA degradation, the amino group of BCAAs is received by α-KG to generate glutamate. Since this transamination is thermodynamically reversible, BCAT-mediated synthesis of BCAAs accompanies the deamination of glutamate. This is important in metabolic regulation, since BCAAs act as signaling molecules which promote mTORC1 activity when their concentrations are elevated [61,62]. Taken together with our previous observations of both elevated TCA cycle enzyme expression and mTORC1 activity in ME/CFS lymphoblasts, these considerations highlight the importance of determining whether ME/CFS cells exhibit altered expression of enzymes involved in BCAA metabolism.

Since BCAT-catalysed transamination is reversible, changes in BCAT expression alone would not indicate the favoured steady-state direction of BCAA metabolism. However, the conversion of BCAAs to branched-chain ketoacids by BCAT is followed by the irreversible production of branched-chain acyl-CoA derivative esters (precursors of TCA cycle intermediates) by the branched-chain ketoacid dehydrogenase (BCKDH) complex in the mitochondria [63]. Since this reaction is irreversible, this represents the first committed and rate-controlling step in the mitochondrial degradation of BCAAs. The expression of BCKDH complex subunits is therefore useful for inferring the favoured steady-state direction of BCAA metabolism.

In both the whole-cell proteomes and transcriptomes, mean expression levels of the cytosolic isoform BCAT1 were not significantly altered in ME/CFS lymphoblasts (Figure 7B). The transcript level of the mitochondrial isoform, BCAT2, was also unaltered (Figure 7B) while the protein was poorly detected in the proteomes. Thus, we found no evidence for changes in the levels of BCAT in ME/CFS cells. However, in the proteomes of ME/CFS lymphoblasts, expression levels of both BCKDH subunits were significantly elevated (BCKDHA levels by 86 ± 27%, *p* = 0.028 and BCKDHB levels by 57 ± 23%, *p* = 0.031) (Figure 7B). At the transcriptional level, BCKDHA showed a slight, non-significant elevation of 13 ± 6%, while BCKDHB levels were significantly elevated by 30 ± 6% (*p* = 0.007) (Figure 7B). This upregulation of both BCKDH complex subunits strongly indicates that BCAAs are also being more heavily utilised to provide the TCA cycle with substrate. In turn this implies elevated degradation of BCAAs by mitochondrial BCAT, necessarily accompanied by increased replenishment of glutamate for utilisation by the GLUD1 or GOT2 routes, both of which are upregulated (Figure 7A).

Within the various pathways through which other amino acids may similarly be utilised, most of the enzymes involved were not detected, or detected at low levels in few samples. The few that were detected were present at relatively low levels compared with those present in other amino acid-degradative processes such as glutaminolysis. This may be related to the reduced accessibility of these alternatives in culture medium and their less preferential metabolic utilisation compared with glutamine [64]. Of those that were detected, the expression of two was significantly altered in ME/CFS lymphoblast proteomes. While their expression was not significantly different from controls at the transcriptional level, the expression levels of GCDH and fumarylacetoacetase (FAH) were significantly elevated in ME/CFS lymphoblast proteomes, with mean levels 74 ± 21% and 61 ± 25% higher than controls, respectively (Figure 7C). As previously noted, GCDH catalyses the reduction of ETF as part of lysine and tryptophan degradation, thereby providing electrons towards OXPHOS [56]. On the other hand, FAH catalyses the final step of phenylalanine degradation, resulting in provision of the TCA cycle intermediate fumarate. Together, both of these enzymes are therefore important for mediating the mitochondrial utilisation of lysine, tryptophan, and phenylalanine as alternative sources of oxidisable substrate. Thus, their elevated expression could reflect increased degradation of these amino acids to assist with driving respiration. In particular, decreased phenylalanine levels were previously reported in ME/CFS patient serum and plasma [4,10], which would be consistent with its increased degradation in such a way.

### 2.6. Expression of Proteasome Subunits Is Elevated in ME/CFS Lymphoblasts

**Figure 8:**
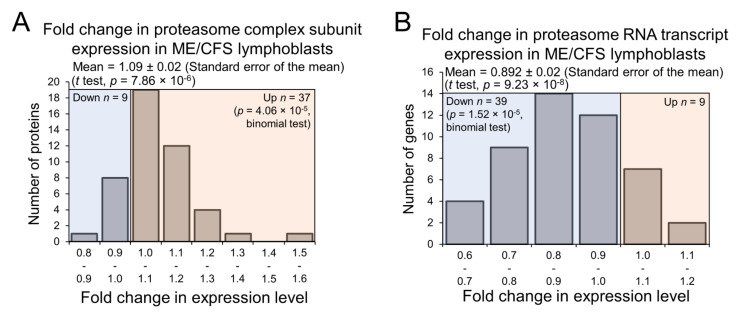
Expression of proteasome complexes in Myalgic Encephalomyelitis/Chronic Fatigue Syndrome (ME/CFS) lymphoblasts is upregulated at the protein level but downregulated at the transcript level. Error bars represent the standard error of the mean. RNA sequencing transcriptomics experiment: ME/CFS *n* = 23, control *n* = 17. Each cell line was sampled once. Mass spectrometry proteomics experiment: ME/CFS *n* = 34, control *n* = 31. Each cell line was sampled once, or twice for a subset of healthy controls arbitrarily selected to act an internal control between experiments in the mass spectrometry proteomics work. (**A**) A total of 46 proteasome complex subunits were detected within the whole-cell proteomes of ME/CFS and control lymphoblasts. Fold change refers to the mean abundance of a given protein in the CFS group divided by the mean abundance in the control group. The fraction of detected proteins that were upregulated (binomial test with H_o_ set to 0.5) and the average extent of the upregulation (single-sample *t* test with H_o_ *m* = 1) were statistically significant. (**B**) A total of 48 RNA transcripts encoding proteasome complex subunits were detected within the whole-cell transcriptomes of ME/CFS and control lymphoblasts. Fold change refers to the mean abundance of a given transcript in the CFS group divided by the mean abundance in the control group. The fraction of detected transcripts that were downregulated (binomial test with H_o_ set to 0.5) and the average extent of the downregulation (single-sample *t* test with H_o_ *m* = 1) were statistically significant.

If ME/CFS lymphoblasts do catabolise such a broad array of amino acids for energy at faster rates in accordance with the elevated expression of these various enzymes, the degradation of cellular proteins could also be affected, since it could constitute an accessible source of free amino acids. This is particularly likely given that lysine, tryptophan, and phenylalanine are all essential amino acids and cannot synthesized de novo in human cells. While following the trend of downregulation of many pathways at the transcriptional level, expression of the proteasome complex subunits was significantly elevated in the proteomes of ME/CFS lymphoblasts compared with controls (Figure 8A,B), implying the upregulation of targeted protein degradation. This was not revealed in the global PANTHER pathway analysis because the proteasomal protein degradation does not feature in the reactome pathways in PANTHER, but is instead treated as a subcellular location. In any case, the upregulated expression of proteasome subunits we observed suggests elevated intracellular protein turnover in ME/CFS lymphoblasts. As a source of free amino acids, this could act to provide the inefficient mitochondria with additional oxidisable substrate. It could also reflect elevated degradation of misfolded proteins naturally accompanying the translational upregulation of many proteins in ME/CFS lymphoblasts.

## 3. Discussion

Our results demonstrate that ME/CFS cells express unchanged levels of glycolytic enzymes but elevated levels of enzymes involved in the pentose phosphate pathway, as well as protein, amino acid and fatty acid degradation. This striking pattern of dysregulated expression of catabolic enzymes provides strong support for previous metabolomics [4,6,10], glycolytic flux [2,8] and mitochondrial function measurements [2] that suggest a metabolic shift towards alternatives to glycolytic provision of oxidisable substrates to the mitochondria. Rather than being mediated by a reduction in glycolytic function, our results support our previous suggestion that this shift is caused by an elevation of alternative catabolic pathways. The observation of an inefficiency in respiratory ATP synthesis by mitochondrial Complex V in ME/CFS cells suggests that this metabolic shift might be compensatory, while the elevated activity of mTORC1 (and possibly AMPK) suggest that it is mediated by cellular stress signaling pathways [2].

A feature of our results is the striking difference in the pattern of expression changes at the RNA and protein levels. The proteomics revealed a broad pattern of elevated expression of proteins involved in alternatives to glycolytic provision and catabolism of oxidisable substrates for mitochondrial respiration. By contrast, the levels of transcripts encoding these proteins were, in many cases, either unchanged or decreased. This is an unexpected but important insight into the underlying cytopathological mechanisms of ME/CFS. It suggests that the overall pattern of dysregulation in ME/CFS cells is a result of a network of normally homeostatic pathways, including competing antagonistic elements like elevated mTORC1 and AMPK activities, that regulate gene expression and metabolism at the transcriptional, translational and posttranslational levels [65]. The major pathways we found to be dysregulated in this way are β-oxidation of fatty acids, glutamine metabolism, branched-chain amino acid catabolism and proteasomal protein degradation.

### 3.1. Preferential Fatty Acid β-Oxidation and Dysregulated Intracellular Energy Stress Signaling

We previously found no changes in glycolytic rate, reserve and capacity in ME/CFS lymphoblasts [2] and here report unaltered expression of glycolytic enzymes at the protein level. Instead of an impaired capacity to undertake glycolysis, our results suggest that changes in ME/CFS lymphoblast metabolism might be driven by dysregulated energy stress signaling and elevated usage of alternatives such as the β-oxidation of fatty acids or increased shunting of glucose towards the PPP. Our observation that enzymes mediating fatty acid β-oxidation are broadly upregulated in ME/CFS lymphoblasts confirms our previous observation in a pilot study [2]. In addition to the other β-oxidation enzymes which we found to be upregulated, more highly expressed enzymes such as very long-chain-specific acyl-CoA dehydrogenase (ACADVL) and acyl-CoA oxidase 1 (ACOX1) suggest elevated VLCFA utilisation. This is consistent with the decreased sphingolipids in ME/CFS patient plasma reported by Naviaux et al. [19], since VLCFA are derived from these. Similarly, elevated fatty acid β-oxidation suggested by Germain et al. is also consistent with our observations here, if the rates of fatty acid β-oxidation are indeed upregulated in accordance with expression of the enzymes involved [10]. Sweetman et al. observed elevated levels of enzymes involved in ketone body metabolism in the proteomes of ME/CFS PBMCs, which could indicate increased oxidation of fatty acids and their derivatives through the TCA cycle [66]. More strikingly within this same study, acyl-CoA dehydrogenases and the beta subunit of mitochondrial trifunctional enzyme (HADHB), specifically, were elevated in their expression in PBMCs [66]—an observation shared in our own work here with lymphoblasts. This is in addition to shared observations of elevated expression of OXPHOS complex subunits—prominently Complexes I and V—and proteins in substrate-providing pathways such as the TCA cycle [66]. Since the Sweetman study examined non-immortalised PBMCs (from which lymphoblasts are derived), this confirms that the upregulated respiratory capacity and upregulation of substrate-providing mitochondrial pathways exhibited by ME/CFS lymphoblasts is present independent of immortalisation.

Despite this broad agreement, there remain some discrepancies in the literature regarding fatty acid β-oxidation. The reduced FAD levels reported by Naviaux et al. and reduced carnitines reported by Nagy-Szakal et al. are interpreted as hindering fatty acid β-oxidation [19,20]. Since these and other previously mentioned studies draw inferences as to cellular function from the levels of blood metabolites, and our work here only investigated expression levels, more direct measures of fatty acid β-oxidation rates in a cellular context should continue to be pursued in the future. One previous study along these lines used Seahorse respirometry and reported unchanged fatty acid utilisation rates in permeabilised PBMCs from ME/CFS patients [67]. However, the metabolic quiescence and greater death rates of ME/CFS lymphocytes [2,68] may have obscured differences, as may have the loss of cytoplasmic context due to permeabilisation. Respiration rates provisioned by fatty acid utilisation were also found to be unchanged in skeletal muscle cells from ME/CFS patients, contrary to the authors’ expectations of elevated, compensatory β-oxidation as part of a shift away from glucose metabolism [9]. The expected increase may have been absent due the reduced exercise that ME/CFS patients can undertake, since exercise upregulates mitochondrial biogenesis and function in muscle [69]. Another possibility is that metabolism by proliferative cells (exemplified by lymphoblasts) and non-proliferative cells (exemplified by muscle cells) in ME/CFS differ in their patterns of substrate utilisation or in their capacity to be metabolically adaptive. Direct assays of fatty acid utilisation rates in lymphoblasts and of protein expression in muscle cells are therefore needed to confirm whether the rates of fatty acid β-oxidation and expression of the involved enzymes are altered in concert with one another in both ME/CFS lymphoblasts and muscle cells.

Accompanying the upregulation of β-oxidation enzymes, we observed a statistically non-significant ~17% increase in the level of phosphorylation of acetyl CoA carboxylase (ACC) in ME/CFS lymphoblasts. If confirmed in future work, this possible increase in AMP-activated protein kinase (AMPK) inhibition of ACC would suggest a shift towards catabolism rather than biosynthesis of fatty acids. On the other hand, mammalian target of rapamycin complex 1 (mTORC1) is known to activate the transcription factor sterol regulatory element-binding protein-1 (SREBP-1) as part of the Akt signaling pathway and to thereby upregulate expression of ACC and fatty acid synthase (FASN) and fatty acid biosynthesis [70,71,72]. Since mTORC1 is chronically hyperactive in ME/CFS lymphoblasts [2], we expected to observe elevated levels of the ACC1 and FASN transcripts. We found that both trended upwards in ME/CFS lymphoblasts but did not reach statistical significance. Nonetheless, the results are consistent with the previously reported elevation of mTORC1 activity [2] and downstream activation of SREBP-1. SREBP-regulated transcription was amongst the pathways we found to be upregulated in the PANTHER analysis of the transcriptomics data (Table 1). Despite this possible upregulation of ACC and FASN transcription, we found no evidence of elevated levels of either protein (proteomics and plate reader assays). If anything, these trended downwards (Figure 6) and, if confirmed in future work, this would also suggest a metabolic shift in favour of fatty acid catabolism.

### 3.2. Dysregulation of Glutamine Metabolism

The elevated expression of enzymes involved in mitochondrial glutamine degradation which we have observed here is consistent with the reductions in blood glutamine levels previously reported in ME/CFS patients [3,4,6]. This strongly suggests elevated usage of glutamine as a mitochondrial substrate by ME/CFS cells. Such dysregulation of glutamine metabolism would have far-reaching consequences given its importance in many cellular processes.

While also serving to replenish metabolic intermediates and reducing equivalents to aid with driving respiration, mitochondrial glutamine degradation itself activates mTORC1 signaling [57,58]. This is thought to occur following glutamate deamination to α-KG by glutamate dehydrogenase (GLUD1) [57], one of the enzymes whose expression we found here is elevated. The α-KG is shuttled into the cytosol by the mitochondrial transporter protein SLC25A11 [73] where it activates mTORC1 via the activation of prolyl hydroxylases [74]. While no members of the prolyl hydroxylase family were detected in the whole-cell proteomics experiments, SLC25A11 was well detected and significantly elevated in its expression (Figure S3). SLC25A11 is one of the mitochondrial transport proteins we previously reported to be upregulated in ME/CFS lymphoblasts [2] and it was also found to be upregulated in PBMCs by Sweetman et al. [66].

If mitochondrial glutamine utilisation is indeed increased as suggested by the elevated expression of the enzymes involved, it could contribute to the chronic hyperactivation of mTORC1 in ME/CFS lymphoblasts. This is particularly likely given the most potent amino acid activator of mTORC1 (leucine) [75] has been proposed to do so specifically by allosteric activation of GLUD1 and upregulation of mitochondrial glutamine catabolism [58]. Further work should be undertaken to clarify the cause-effect relationships involved in mTORC1 activation in ME/CFS lymphoblasts.

Increased glutamate flux to aspartate catalysed by GOT2 is suggested in our results by the elevation of mitochondrial aspartate aminotransferase (GOT2) expression and has been proposed in metabolomic studies by others [4]. As earlier described, this mechanism is an important component of the malate-aspartate shuttle (MAS) which balances cytosolic and mitochondrial redox status. In ME/CFS lymphoblasts, the cytoplasmic enzyme malate dehydrogenase (MDH1) which is critical in the MAS was also elevated in its expression (Figure S3). While the MAS is important for providing reducing equivalents for OXPHOS, its functions are also necessary to facilitate other dysregulated processes which provide ME/CFS mitochondria with oxidisable substrate, such as peroxisomal fatty acid β-oxidation. Peroxisomal β-oxidation generates NADH and is sustainable when mitochondrial shuttling mechanisms are available to oxidise NADH back to NAD^+^ [46]. Increased MAS activity in ME/CFS lymphoblasts would therefore act not only to support respiration directly by the replenishment of mitochondrial reducing equivalents, but would also indirectly assist with providing the TCA cycle with acetyl-CoA derived from alternative sources such as VLCFA.

The elevated expression we observed in ME/CFS lymphoblasts adds to the accumulating body of evidence from proteomics and metabolomics supporting hypercatabolism of glutamine in ME/CFS cells, so direct assays of glutamine utilisation rates in ME/CFS lymphoblasts or other metabolically active cell types are warranted to confirm this. It will be important to assess this in actively metabolising, proliferative cells given that prior work by others found no difference in glutamine-assisted respiration in metabolically quiescent, permeabilised PBMCs and a small sample of permeabilised myotubes [67]. Since conventional mammalian cell culture medium is supplemented with glutamine in physiological abundance to satisfy proliferative cell metabolism programmes [76], it would also be useful to test the effects of varying glutamine availability in culture on ME/CFS and control lymphoblasts.

Since glutamine-derived α-KG is critical for the induction of epigenetic modifications by DNA and histone demethylases [77], increased usage of glutamine and TCA cycle intermediates to drive mitochondrial respiration could be associated with changes in the levels of DNA and histone methylation. This could be important given the large number of upregulated and even larger number of downregulated transcripts in the ME/CFS lymphoblast transcriptomes. Multiple studies examining DNA methylation in ME/CFS patients have taken place in recent years [78,79,80,81,82]. These studies examining differential methylation status have been recently reviewed, with the proportions of differentially hypo- or hypermethylated sites in ME/CFS being inconsistent across studies [83]. More recently, Helliwell et al. conducted the first epigenetic study to employ reduced representation bisulphite sequencing, which is capable of greater CpG site coverage than previous array based methods [84]. This study found significant differences in similar numbers of both hypo- and hypermethylated sites in the genomes of PBMCs from ME/CFS patients, suggesting that significant epigenetic dysregulation is present but does not specifically favour hypo- or hypermethylation. This contrasts with our finding of significantly more downregulated than upregulated transcripts in ME/CFS lymphoblasts.

Within the regulatory regions of protein-coding genes investigated in the Helliwell study, it is worthwhile noting that the gene encoding the Complex 1 subunit NDUFA11 was hypomethylated [84]. This is consistent with the elevated Complex 1 expression evident in this and our previous study of lymphoblasts [2], as well as the same authors’ study of PBMCs [66]. Continued epigenetic studies utilising different cell types would be valuable, since it may be possible that in proliferative cell types such as lymphoblasts, factors such as altered gene expression programmes or glutamine depletion are accentuated and thus may impact DNA methylation status to a greater degree than in PBMCs.

### 3.3. Dysregulated Branched-Chain Amino Acid- and Protein-Degradative Pathways

Upregulation of the branched-chain ketoacid dehydrogenase (BCKDH) complex in ME/CFS lymphoblasts strongly indicates elevated mitochondrial catabolism of branched-chain amino acids (BCAAs) as a source of oxidisable substrate and TCA cycle intermediates. It has been shown in mice that mTORC1 function is essential for stimulating BCKDH degradation of BCAAs, since treatment with the mTORC1 inhibitor rapamycin suppressed activation of the BCKDH complex [59]. Since mTORC1 is hyperactive in ME/CFS lymphoblasts, we expected and indeed observed elevated expression of BCKDH in the ME/CFS proteomes. However, the increased degradation and depletion of BCAA that this would produce could in turn constrain the activity of mTORC1, since elevated cellular BCAA concentrations activate mTORC1 [62]. It is seems likely that mTORC1 activity upregulates BCAA degradation while mTORC1 itself is activated by other phenomena in ME/CFS lymphoblasts, such as the increased mitochondrial degradation of glutamine.

BCKDH activity is also regulated by inhibitory phosphorylation by its kinase BCKDK [85], but the levels of BCKDK in ME/CFS lymphoblasts were not significantly different from those of healthy controls (Figure S3). It would be valuable to directly measure the rates of BCAA utilisation in ME/CFS lymphoblasts to confirm the directional shift in mitochondrial BCAA metabolism that is indicated by elevated BCKDH expression.

A source of BCAAs and other amino acids, both as oxidisable substrates for the mitochondria and as activators of mTORC1 is proteolysis. Proteolysis mediated by the ubiquitin-proteasome system results in the targeted degradation of intracellular proteins and the release of free amino acids. It is a normal response to insufficient caloric intake and is dysregulated in many diseases [86]. The elevated expression of proteasome subunits and assembly factors in ME/CFS lymphoblasts suggests that targeted protein degradation is upregulated. The recent proteomic study by Sweetman et al. [66] also reported elevated expression of proteasome subunits and assembly factors in ME/CFS PBMCs. Together with our results, this provides compelling evidence that the ubiquitin-proteasome system is upregulated in lymphoid cells from ME/CFS patients. This would not only act to provide free amino acids, but may also be necessary to degrade the higher number of misfolded proteins which would naturally accompany the translational upregulation of many proteins in ME/CFS lymphoblasts. Since enzymes involved in the autophagic degradation of proteins were poorly detected in the whole-cell proteomes, it is uncertain whether the degradation of intracellular proteins by autophagy is also upregulated.

Autophagy is stimulated by activation of serine/threonine protein kinase (Ulk1) by AMPK, and inhibited by mTORC1, while mTORC1 itself is inhibited by AMPK activity [87]. However, concurrent activation of AMPK and mTORC1 can still occur and result in sustained autophagy [88,89]. This could meet the need to degrade the increased number of misfolded proteins which naturally accompany the upregulated translation of proteins that is stimulated by mTORC1 [90,91], or to catabolically replenish metabolic intermediates used to support proliferative cell metabolism [92], such as those utilised in the TCA cycle. Given that the elevation of mean AMPK activity levels did not reach statistical significance, and that this occurred alongside elevated mTORC1 activity in ME/CFS lymphoblasts [2], it is unclear whether to expect autophagy to be increased or decreased in these cells. Potential dysregulation of autophagy in ME/CFS therefore requires future study.

## 4. Materials and Methods

### 4.1. Participant Cohort Recruitment, Composition and Subsets

All participants were of European descent and belonged to two clinical groups: ME/CFS patients diagnosed according to the Canadian Consensus Criteria [93] and healthy controls without any family history of ME/CFS or similar myalgias, nor cohabiting with ME/CFS patients. Prospective recruits with other known reasons for overlapping symptoms were excluded. A 15 mL blood sample was taken per participant in heparin-treated vacutainer tubes for PBMC isolation and subsequent lymphoblast preparation. Blood collections were undertaken by trained staff at CFS Discovery Clinic in Melbourne, Australia prior to its closure, after which trained staff undertook blood draws in the homes of severely ill patients unable to travel, or at a dedicated facility at La Trobe University, Melbourne, Australia. Informed written consent was obtained from all subjects involved in the study.

Cell lines were randomly selected from our library to be utilised in these experiments to avoid selection bias. Sample sizes varied between experiments due to cost factors. Details of the lymphoblast subsets used are as follows:

#### 4.1.1. Proteomics

This subset included 34 ME/CFS patients (88% female, median age 52.5, age range 26–71) and 31 controls (45% female, median age 30, age range 19–58). The difference in gender proportions (Fisher’s exact test *p* = 0.0004) and the distribution of ages (Fisher’s exact test, *p* = 0.000183 using 15 year bins) were statistically significant. However, there was no significant effect of either age (multiple regression) or gender (ANOVA) on analysed experimental outcomes in either patients or controls (*p* > 0.05).

#### 4.1.2. Transcriptomics

This subset included 23 ME/CFS patients (82% female, median age 52, age range 27–71) and 17 controls (59% female, median age 41, age range 21–58). The difference in gender proportions (Fisher’s exact test *p* = 0.153) and the distribution of ages (Fisher’s exact test, *p* = 0.102 using 15 year bins) were not statistically significant. There was also no effect of either age (multiple regression) or gender (ANOVA) on analysed experimental outcomes in either patients or controls (*p* > 0.05).

#### 4.1.3. AMPK Activity Assay

This subset included 28 ME/CFS patients (79% female, median age 42, age range 22–67) and 24 controls (58% female, median age 33, age range 21–58). The difference in gender proportions (Fisher’s exact test *p* = 0.141) and the distribution of ages (Fisher’s exact test, *p* = 0.474 using 15 year bins) were not statistically significant. There was also no effect of either age (multiple regression) or gender (ANOVA) on analysed experimental outcomes in either patients or controls (*p* > 0.05).

### 4.2. Immortalisation, Growth, Storage and Counting of Lymphoblast Cell Lines

Lymphoblast cell lines were immortalised, cultured, cryopreserved or counted as previously described [2,94]. The immortalisation procedure is briefly described here. Lymphocytes were isolated by Ficoll–Paque density centrifugation and counted. Next, 5 × 10^6^ cells were harvested for immortalisation and were resuspended in 5 mL RPMI 1640 without l-glutamine (Life Technologies, Carlsbad, CA, USA) supplemented with 1× Glutamax (Life Technologies, Carlsbad, CA, USA), 10% FBS and 1% Penicillin/Streptomycin. Excess lymphocytes were separated into aliquots of 5 × 10^6^ cells, harvested and resuspended in 250 μL of Recovery™ Cell Culture Freezing Medium (Life Technologies, Carlsbad, CA, USA) and stored at −80 °C.

For immortalisation, 1 mL culture supernatant from B95.8 cells expressing Epstein–Barr virus was added, and 150 µL of the mix was seeded per well in a 96-well U-bottom plate, then incubated for one hour within a humidified 5% CO_2_ incubator at 37 °C. A final concentration of 500 ng/mL Cyclosporin A (Sigma-Aldrich, St. Louis, MO, USA) was then added to each well. Cultures were fed weekly by replacing half of the medium with the same formulation, without disturbing the cells. This process was repeated over a period of approximately three weeks until the cells were confluent and growing rapidly, after which the lymphoblast cultures were transferred to T25 flasks in Minimum Essential Medium α (Life Technologies, Carlsbad, CA, USA) supplemented with 10% FBS and 1% Penicillin/Streptomycin to be used for experiments and to be cryopreserved.

### 4.3. Whole-Cell Proteome Analysis

Each sample (3 × 10^6^ lymphoblasts in 100 µL PBS) was prepared for subsequent proteome analysis by the La Trobe University Comprehensive Proteomics Platform according to the following protocol:

Each sample was dried using a SpeedVac Concentrator and Savant Refrigerated Vapor trap (Thermo-Fisher Scientific, Waltham, MA, USA). Samples were resuspended in 8 M urea, 100 mM tris pH = 8.3. A volume of 1 µL of tris (2-carboxyethyl) phosphine hydrochloride (TCEP, 200 mM solution in water) was then added to the samples and incubated overnight at 21 °C in a ThermoMixer (Eppendorf AG, Hamburg, Germany). Four microliters of 1 M iodoacetamide (IAA in water) was added the following day and incubated in the dark at 21 °C. Next, 500 µL of 50 mM tris (pH 8.3) and 1 μg trypsin was added to samples and left for 6 h at 37 °C in an incubator. Another 1 μg trypsin was added for double digestion and incubated overnight at 37 °C. The digested samples were purified for mass spectrometry analysis prior to peptide reconstitution and separation using Sep-Pak light C18 cartridges (Waters, Milford, CT, USA) according to manufacturer standard procedures. Data were collected on a Q Exactive HF (Thermo-Fisher Scientific, Waltham, MA, USA) in the Data-Dependent Acquisition mode using *m*/*z* 350–1500 as mass spectrometry (MS) scan range at 60,000 resolution, HCD MS/MS spectra were collected for the 15 most intense ions per MS scan at 15,000 resolution with a normalised collision energy of 28% and an isolation window of 1.4 *m*/*z*. Dynamic exclusion parameters were set as follows: exclude isotope on, duration 30 s and peptide match preferred. Other instrument parameters for the Orbitrap were MS maximum injection time 30 ms with AGC target 3 × 106, for a maximum injection time of 25 ms with AGT target of 1 × 105. Raw files consisting of high-resolution MS/MS spectra were processed with MaxQuant v. 1.6.1.0 to detect features and identify proteins using the search engine Andromeda. UniProtKB/Swiss-Prot *Homo sapiens* sequence data were used as the database for the search engine. To assess the false discovery rate (FDR) a decoy dataset was generated by MaxQuant after reversing the sequence database. Theoretical spectra were generated using the enzyme as trypsin allowing two missed cleavages. The minimum required peptide length used was seven amino acids. Carbamidomethylation of Cys was set as a fixed modification, while *N*-acetylation of proteins and oxidation of Met were set as variable modifications. Precursor mass tolerance was set to 5 ppm and MS/MS tolerance to 0.05 Da. The “match between runs” option was enabled in MaxQuant to transfer identifications made between runs on the basis of matching precursors with high mass accuracy. PSM and protein identifications were filtered using a target-decoy approach at a false discovery rate (FDR) of 1%.

### 4.4. RNA Extraction from Lymphoblasts

At least 1 x 10^6^ lymphoblasts were harvested by centrifugation at 500× *g* for 5 min and lysed promptly with 1 mL Purezol RNA Isolation Reagent (Bio-Rad, Hercules, CA, USA) in a microcentrifuge tube. A volume of 200 µL chloroform was added to each tube, mixed well and incubated at room temperature for 15 min. This mixture was centrifuged at 12,000× *g* for 15 min at 4 °C and the colourless, top-layer aqueous phase transferred to a fresh tube. A volume of 500 µL isopropanol was added to each tube and vortexed for 5 s, then incubated at RT for 10 min. The supernatant was then carefully discarded following a 12,000× *g* centrifugation for 8 min at 4 °C. The RNA was then washed with 1 mL 75% ethanol at 7500× *g* for 5 min at 4 °C, the supernatant removed and the tube briefly air-dried. The sedimented RNA was then dissolved at 50 µL RNAse-free water. This was then treated with the RQ1 DNAse protocol (Promega, Madison, WI, USA) at working concentrations as specified by the manufacturer, for 60 min at 37 °C before the reaction was terminated by addition of the “stop” solution. RNA was then used for required assays on the same day or stored at −80 °C until used.

### 4.5. RNA Sequencing

RNA samples were prepared according to the foregoing protocol and sent to Australian Genome Research Facility (AGRF), Melbourne on dry ice for mRNA sequencing and quantification. This was achieved using the Illumina TruSeq stranded mRNA protocol as per manufacturer instructions (Illumina, Inc. San Diego, CA, USA).

### 4.6. Confirmatory Semi-Quantitative Western Blotting

Cells were lysed in 2× Laemmli sample buffer with complete protease inhibitor cocktail (Roche, Basel, Switzerland). A small aliquot of each sample was briefly sonicated and analysed for total protein concentration using a Qubit Protein Assay Kit and Qubit 2.0 Fluorometer (Thermo-Fisher Scientific, Waltham, MA, USA) according to manufacturer instructions.

The samples were then heated to 90 °C for 10 min and 30 µg of total protein was loaded into each well in 10% SDS polyacrylamide stain-free gels. After electrophoresis, proteins were transferred onto polyvinylidene difluoride (PVDF) membranes using a Mini gel tank with the Mini Blot module (Thermo-Fisher Scientific, Waltham, MA, USA) 60 min at 10 V, 300A, at 4 °C and then blocked for 1 h with blocking buffer (1% casein, TBST buffer) and incubated overnight with primary antibodies diluted 1:1000 in blocking buffer. The antibodies used were ACO2 (D6D9, cat #6571 Cell Signaling Technology, Danvers, MA, USA) SdhA (D6J9M, cat #11998, Cell Signaling Technology, Danvers, MA, USA), and CPTC-MDH1-1 developed by Clinical Proteomics Technologies for Cancer, obtained from the Developmental Studies Hybridoma Bank (Department of Biology, The University of Iowa, Iowa City, IA, USA). Stain-free gel scans were utilised as the internal loading control in combination with an HRP-conjugated secondary antibody for detection (Thermofisher Scientific, donkey anti-rabbit IgG, cat #A16023, and Goat anti-mouse IgG, cat #31430). Following incubation with antibodies, the membranes were washed three times with TBS buffer containing 0.5% Tween 20, and visualised using a chemiluminescent substrate (Clarity Western ELC substrate, Bio-Rad Hercules, CA, USA) and the Amersham Imager 600 (GE Healthcare Life Sciences, Chicago, IL, USA) and analysed using the Image Lab software (Bio-Rad, Hercules, CA, USA). One arbitrarily selected control cell line was included in every blot as internal normalisation control for between-experiment variation.

### 4.7. Confirmatory qRT-PCR

SYBR green fluorescence was used to quantify fragments of transcripts of interest, using the following primers: GLS-Forward (5′ GGAAGCCTGCAAAGTAAACCC 3′), GLS-Reverse (5′ CCAAAGTGCAGTGCTTCATCC 3′) or SdhB-Forward (5′ ACTCTAGCTTGCACCCGAAG 3′) and SdhB-Reverse (5′ GCTGCTTGCCTTCCTGAGAT 3′). A fragment of the transcript encoding histone gene *HIST1H1C* was amplified alongside the investigated genes as an internal control, using the following primers: Forward (5’ GCGGCGCAACTCCGAAGAAG 3’) and Reverse (5’ AGCGGCCTTGGGCTTCACAG 3’).

The reaction mixture was prepared according to manufacturer instructions (iTaq Universal One-Step Kit, Bio-Rad, Hercules, CA, USA), primers at a concentration of 500 nM, and sample RNA constituting 1/10 of the reaction mixture. Negative control wells with no sample were included to confirm that no contamination was present. The CFX Connect Real-Time PCR Detection System (Bio-Rad, Hercules, CA, USA) was used to amplify and detect the transcript fragments of interest. The cycle threshold obtained for the target transcript fragments of interest was subtracted from that of HIST1H1C to assess relative quantification.

### 4.8. AMPK Activity Assay (ACC1/2 Phosphorylation State)

AMPK activity in ME/CFS lymphoblast lysates was measured using a time-resolved FRET-based multiwell plate assay of the phosphorylation state of ACC1/2 (Cisbio Bioassays, Codolet, France). Cells were harvested, resuspended in growth medium at 1.2 ×10^6^ cells/mL and plated in six replicates at 3 × 10^4^ cells/well in a 384-well plate. Cells were incubated at 5% CO_2_/37 °C for 4 h, with two of the replicates subjected to AMPK inhibition by 30 µM SBI-0206965 (Selleckchem, Houston, TX, USA) and two of the replicates subjected to AMPK activation by 30 µM A-769662 (AdooQ Bioscience, Irvine, CA, USA). The remaining two replicates were treated with an equivalent concentration of DMSO as a control. Lysis buffer was added to each well as per manufacturer instructions and the plate mixed on an orbital shaker for 40 min before plating each sample into a 384 well white plate—incorporating various controls and antibody mix (anti-ACC (Ser79) antibody labelled with d2 acceptor, and anti-phospho-ACC (Ser79) antibody labelled with Eu3+-cryptate donor) according to manufacturer instructions. After a 24 h incubation at RT, the plate was scanned by the Clariostar plate reader (BMG Labtech, Ortenberg, Germany) and the ratio of the FRET signal from anti-phospho-ACC (Ser79) antibody to the donor fluorescence signal from anti-ACC (Ser79) antibody was measured according to instructions. Lymphoblasts from an arbitrarily selected control cell line were included in each experiment as an internal control for between-experiment variation.

### 4.9. Statistical Analysis

Data were analysed using Microsoft Excel with the Winstat add-in (http://www.winstat.com, downloaded 1 March 2018) or R using the packages R Commander [95], REzy [96], Rattle [97], pROC [98], edgeR [99,100] and stats. Unless otherwise specified, two-sample tests used the Welch t test. ANOVA and Fisher’s exact tests were used as specified and appropriate. The significance of individual coefficients in multiple regression analysis was tested using *t* tests.

Proteomics data were analysed employing the software Scaffold (Proteome Software) prior to exporting to Excel for additional analysis. Proteins detected in fewer than 5 samples were excluded from the analysis. Intensity-Based Absolute Quantitation abundance values were normalised to the mean total abundance from 5 healthy controls which were arbitrarily selected for inclusion in each proteomics experiment to control for any between-experimental variation. Transcriptomics data were initially analysed by AGRF using the edgeR package to perform genewise negative binomial generalised linear models with quasi-likelihood tests and gender as a batch effect. For individual transcripts, read counts were normalised to counts per million mapped reads within each respective sample. Data were exported to Excel for additional analysis.

PANTHER over-representation tests [27,28,29] were carried out in the early stages of analysis in order to obtain an objective, broad perspective of the data to inform unbiased subsequent analysis of individual, differentially regulated pathways. To facilitate this, genes/proteins were assigned a Q value to correct for multiple comparisons according to the Benjamini–Hochberg method, and separated into two lists: those significantly up- or downregulated. Each list was entered into the PANTHER over-representation tool, binomial tests were selected as the statistical test and the resulting lists exported to Excel for further assessment. The list of transcripts or proteins detected in the entire experiment was uploaded as the reference list used by PANTHER to determine the number of “expected” pathway hits for a given number of transcripts or proteins.

In closer subsequent analysis of individual pathways, detected proteins or transcripts were identified as belonging to a single functional group (e.g., TCA cycle) or respiratory complex using the NCBI gene ontology (GO) annotation database [101] and manually curated for relevance to the group of interest (occasional erroneous inclusions were removed). The binomial test of proportions was employed to assess whether all detected proteins or transcripts in a single functional group or respiratory complex were together altered in their frequency of up- or downregulation in the ME/CFS group compared to controls, the null hypothesis being that the levels of each protein or transcript had an equal probability of departing in either direction from the control average. For functional groups of proteins where we possessed prior experimental evidence of upregulation, the alternative hypothesis was that the levels of each protein were expected to be greater than the control average. Single-sample *t* tests were also used to assess whether the average fold change in the levels of all detected proteins or transcripts in a single functional group or enzyme complex in the ME/CFS cohort was significantly different from the normalised healthy control mean, or expected to be greater than the control mean for those proteins which we possessed prior evidence of upregulation.

## 5. Conclusions

This study aimed to further examine pathways which provide oxidisable substrate to the mitochondria in ME/CFS lymphoblasts. Our results here have confirmed our previous observations of elevated OXPHOS complex, TCA cycle enzyme, fatty acid β-oxidation enzyme and mitochondrial solute carrier expression in ME/CFS lymphoblasts. Unchanged levels of glycolytic enzymes are consistent with the unchanged rates of glycolysis that we previously observed in these cells. Our new observations also demonstrate upregulated expression of enzymes involved in the PPP and mitochondrial degradation of amino acids for energy. Together, these findings strengthen the proposal that ME/CFS lymphoblasts increasingly utilise alternatives to glycolysis in an attempt to compensate for the respiratory inefficiency by Complex V which we previously reported. Many of the pathways or proteins upregulated in our proteomic dataset overlap with recent work by others using non-immortalised PBMCs [66], strongly indicating that these changes are inherent to lymphoid cells from ME/CFS patients. Combined analysis with our transcriptomic dataset indicates the upregulated expression of many mitochondrial proteins at the translational level, likely stimulated by mTORC1 signaling. Our early exploratory analysis of both datasets also indicated the broad activation of immunological pathways in ME/CFS lymphoblasts, which provides a basis for future hypotheses and examination. In addition, we previously found that parameters of lymphoblast mitochondrial function, mTORC1 signaling and PBMC viability could accurately distinguish ME/CFS cases from controls [68]. The 259 proteins and 2243 transcripts which we found to be significantly altered in expression after correction for multiple comparisons therefore highlight the possibility that diagnostic panels of differentially expressed genes may be successfully deployed to similar ends in future biomarker discovery work.

## Figures and Tables

**Table 1:** Significantly altered mitochondrial, substrate-providing and related metabolic pathways of interest as indicated by PANTHER gene expression analysis of both the proteomic and transcriptomic datasets. “Reactome Pathway” was set as the level of biological granularity. Pathways were categorized and colour-coded by areas of most interest to this investigation as follows: green for carbohydrate metabolism, purple for the TCA cycle and respiration, blue for lipid metabolism, red for amino acid metabolism, yellow for protein degradation, cyan for other mitochondrial, and grey for transport of substrate molecules.

Reactome Pathway	Category	Dataset	Altered Fraction	Fold Enriched	Binomial Test *p*-Value
Gluconeogenesis (R-HSA-70263)	Carbohydrate metabolism	Proteomics	Upregulated	3.81	0.022
Pentose phosphate pathway (R-HSA-71336)	Carbohydrate metabolism	Proteomics	Upregulated	4.52	0.03
Citric acid (TCA) cycle and respiratory electron transport (R-HSA-1428517)	TCA cycle and respiration	Proteomics	Upregulated	1.89	0.027
Formation of ATP by chemiosmotic coupling (R-HSA-163210)	TCA cycle and respiration	Proteomics	Upregulated	4.52	0.03
Respiratory electron transport, ATP synthesis by chemiosmotic coupling, and heat production by uncoupling proteins. (R-HSA-163200)	TCA cycle and respiration	Transcriptomics	Downregulated	3.59	1.33 × 10^−11^
Respiratory electron transport (R-HSA-611105)	TCA cycle and respiration	Transcriptomics	Downregulated	3.69	2.14 × 10^−10^
Citric acid (TCA) cycle and respiratory electron transport (R-HSA-1428517)	TCA cycle and respiration	Transcriptomics	Downregulated	2.83	1.95 × 10^−9^
Complex I biogenesis (R-HSA-6799198)	TCA cycle and respiration	Transcriptomics	Downregulated	4.19	7.59 × 10^−8^
Beta-oxidation of lauroyl-CoA to decanoyl-CoA-CoA (R-HSA-77310)	Lipid metabolism	Proteomics	Upregulated	13.57	0.0015
Beta-oxidation of hexanoyl-CoA to butanoyl-CoA (R-HSA-77350)	Lipid metabolism	Proteomics	Upregulated	10.85	0.0028
Beta-oxidation of octanoyl-CoA to hexanoyl-CoA (R-HSA-77348)	Lipid metabolism	Proteomics	Upregulated	10.85	0.0028
Beta-oxidation of decanoyl-CoA to octanoyl-CoA-CoA (R-HSA-77346)	Lipid metabolism	Proteomics	Upregulated	10.85	0.0028
Mitochondrial fatty acid beta-oxidation of unsaturated fatty acids (R-HSA-77288)	Lipid metabolism	Proteomics	Upregulated	10.85	0.0028
Fatty acid metabolism (R-HSA-8978868)	Lipid metabolism	Proteomics	Upregulated	2.87	0.0029
Acyl chain remodeling of CL (R-HSA-1482798)	Lipid metabolism	Proteomics	Upregulated	18.09	0.0057
Beta-oxidation of myristoyl-CoA to lauroyl-CoA (R-HSA-77285)	Lipid metabolism	Proteomics	Upregulated	18.09	0.0057
Mitochondrial fatty acid beta-oxidation (R-HSA-77289)	Lipid metabolism	Proteomics	Upregulated	5.51	3.32 × 10^−4^
Beta-oxidation of palmitoyl-CoA to myristoyl-CoA (R-HSA-77305)	Lipid metabolism	Proteomics	Upregulated	18.09	6.64 × 10^−4^
Mitochondrial fatty acid beta-oxidation of saturated fatty acids (R-HSA-77286)	Lipid metabolism	Proteomics	Upregulated	10.34	6.72 × 10^−4^
NR1H2 and NR1H3 regulate gene expression linked to triglyceride lipolysis in adipose (R-HSA-9031528)	Lipid metabolism	Transcriptomics	Upregulated	7.47	0.03
Regulation of cholesterol biosynthesis by SREBP (SREBF) (R-HSA-1655829)	Lipid metabolism	Transcriptomics	Upregulated	2.3	0.026
Regulation of lipid metabolism by PPARalpha (R-HSA-400206)	Lipid metabolism	Transcriptomics	Upregulated	1.87	0.031
Phenylalanine and tyrosine metabolism (R-HSA-8963691)	Amino acid metabolism	Proteomics	Upregulated	7.24	0.032
Glutamate neurotransmitter release cycle (R-HSA-210500)	Amino acid metabolism	Proteomics	Upregulated	13.57	0.0015
Neurotransmitter release cycle (R-HSA-112310)	Amino acid metabolism	Proteomics	Upregulated	6.03	0.014
Metabolism of amino acids and derivatives (R-HSA-71291)	Amino acid metabolism	Proteomics	Upregulated	1.64	0.021
Aspartate and asparagine metabolism (R-HSA-8963693)	Amino acid metabolism	Proteomics	Upregulated	7.24	0.032
Lysine catabolism (R-HSA-71064)	Amino acid metabolism	Proteomics	Upregulated	6.03	0.044
Metabolism of amino acids and derivatives (R-HSA-71291)	Amino acid metabolism	Transcriptomics	Downregulated	3.13	2.06 × 10^−20^
Response of EIF2AK4 (GCN2) to amino acid deficiency (R-HSA-9633012)	Amino acid metabolism	Transcriptomics	Downregulated	5.85	6.10 × 10^−26^
Ubiquitin-dependent degradation of Cyclin D (R-HSA-75815)	Protein degradation	Transcriptomics	Downregulated	2.93	2.75 × 10^−4^
Autodegradation of the E3 ubiquitin ligase COP1 (R-HSA-349425)	Protein degradation	Transcriptomics	Downregulated	2.93	2.75 × 10^−4^
Vpu mediated degradation of CD4 (R-HSA-180534)	Protein degradation	Transcriptomics	Downregulated	2.93	2.75 × 10^−4^
Ubiquitin-mediated degradation of phosphorylated Cdc25A (R-HSA-69601)	Protein degradation	Transcriptomics	Downregulated	2.93	2.75 × 10^−4^
Degradation of GLI2 by the proteasome (R-HSA-5610783)	Protein degradation	Transcriptomics	Downregulated	2.79	2.98 × 10^−4^
GLI3 is processed to GLI3R by the proteasome (R-HSA-5610785)	Protein degradation	Transcriptomics	Downregulated	2.79	2.98 × 10^−4^
Degradation of GLI1 by the proteasome (R-HSA-5610780)	Protein degradation	Transcriptomics	Downregulated	2.75	3.60 × 10^−4^
Processing of SMDT1 (R-HSA-8949664)	Other mitochondrial	Proteomics	Upregulated	6.78	0.01
Release of apoptotic factors from the mitochondria (R-HSA-111457)	Other mitochondrial	Proteomics	Upregulated	12.06	0.012
Mitochondrial biogenesis (R-HSA-1592230)	Other mitochondrial	Proteomics	Upregulated	2.81	0.013
Transcriptional activation of mitochondrial biogenesis (R-HSA-2151201)	Other mitochondrial	Proteomics	Upregulated	3.45	0.03
Mitochondrial translation initiation (R-HSA-5368286)	Other mitochondrial	Transcriptomics	Downregulated	3.37	1.80 × 10^−8^
Mitochondrial translation termination (R-HSA-5419276)	Other mitochondrial	Transcriptomics	Downregulated	3.37	1.80 × 10^−8^
Mitochondrial translation (R-HSA-5368287)	Other mitochondrial	Transcriptomics	Downregulated	3.26	2.23 × 10^−8^
Mitochondrial protein import (R-HSA-1268020)	Other mitochondrial	Transcriptomics	Downregulated	2.64	3.73 × 10^−4^
Mitochondrial translation elongation (R-HSA-5389840)	Other mitochondrial	Transcriptomics	Downregulated	3.48	5.03 × 10^−9^
Mitochondrial calcium ion transport (R-HSA-8949215)	Other mitochondrial	Proteomics	Upregulated	6.96	8.53 × 10^−4^
Transport of nucleotide sugars (R-HSA-727802)	Transport of substrate molecules	Transcriptomics	Upregulated	4.98	0.023
SLC transporter disorders (R-HSA-5619102)	Transport of substrate molecules	Transcriptomics	Upregulated	2.03	0.048
Transport of small molecules (R-HSA-382551)	Transport of substrate molecules	Transcriptomics	Upregulated	1.35	0.037
Signaling by Leptin (R-HSA-2586552)	Metabolism	Proteomics	Upregulated	9.05	0.021
Diseases of metabolism (R-HSA-5668914)	Metabolism	Proteomics	Upregulated	3.02	0.045
Metabolism (R-HSA-1430728)	Metabolism	Proteomics	Upregulated	1.51	2.12 × 10^−4^
Activation of gene expression by SREBF (SREBP) (R-HSA-2426168)	Metabolism	Transcriptomics	Upregulated	2.3	0.049
Metabolism (R-HSA-1430728)	Metabolism	Transcriptomics	Downregulated	1.43	3.80 × 10^−8^

**Table 2:** Elevated expression of oxidative phosphorylation complex subunits, TCA cycle enzymes and SLC25 family transporters in whole-cell proteomes from Myalgic Encephalomyelitis/Chronic Fatigue Syndrome patient lymphoblasts (*n* = 34) compared to healthy controls (*n* = 31) was replicated from previous work. Each cell line was sampled once, or twice for a subset of healthy controls arbitrarily included to act as an internal control across each experiment in the proteomics work. Proteins detected in fewer than five samples were excluded. Fold change refers to the mean abundance of a given protein in the CFS group divided by its mean abundance in the control group, with the initial relative abundance determined by normalising Intensity-Based Absolute Quantitation abundance to the internal control average within the respective experiment. Binomial tests were employed to assess fraction upregulated with H_o_ set to *p =* 0.5 (equal up- and downregulated proportions) and H_1_ being that the upregulated proportion was greater. Single-sample *t* tests were employed to assess magnitude of upregulation with H_o_ as mean fold change ≤1 and H_1_ as mean fold change >1.

Functional Category	Number of Subunits Detected	Fraction Fold Change >1 in ME/CFS Proteomes	Binomial Test *p* Value	Mean Fold Change (± Standard Error)	Single-Sample *t* Test *p* Value
Complex I	23	17/23	0.017	1.20 ± 0.05	3.44 × 10^−4^
Complex II	2	2/2	NA	1.08 ± 0.03	NA
Complex III	6	6/6	0.16	1.22 ± 0.03	7.69 × 10^−4^
Complex IV	10	7/10	0.17	1.07 ± 0.04	0.07
Complex V	22	18/22	2.17 × 10^−3^	1.11 ± 0.02	1.18 × 10^−4^
All 5 Complexes	63	50/63	1.51 × 10^−6^	1.14 ± 0.02	1.21 × 10^−8^
TCA Cycle	19	18/19	3.82 × 10^−5^	1.17 ± 0.04	1.03 × 10^−4^
Protein import complex subunits	11	4/11	0.89	1.00 ± 0.04	0.81
SLC25 family	11	9/11	0.033	1.33 ± 0.14	0.016

**Table 3:** Expression of transcripts encoding subunits of the translocase of the inner mitochondrial membrane (TIMM), translocase of the outer membrane (TOMM) and sorting and assembly machinery (SAMM) mitochondrial protein import complexes as well as that oxidative phosphorylaton complexes I, III, IV, and V was reduced in lymphoblasts from Myalgic Encephalomyelitis/Chronic Fatigue Syndrome (ME/CFS) patients (*n* = 23) compared to healthy controls (*n* = 17). Conversely, expression of Complex II transcripts was elevated, while the expression of transcripts encoding TCA cycle enzymes and SLC25 family transporters were unchanged. Each cell line was sampled once in an RNA sequencing transcriptomics experiment. Mean fold change was calculated for the ME/CFS group versus the control average for each transcript. Binomial tests were employed to assess the fraction differentially expressed with H_o_ set to 0.5. Single-sample *t* tests were employed to assess magnitude of differential expression with H_o_ being mean fold change = 1.

Functional Category	Number of Transcripts Detected	Number of Transcripts with Fold Change <1	Number of Transcripts with Fold Change >1	Binomial Test *p* Value	Mean Fold Change (± Standard Error)	Single-Sample *t* Test *p* Value
Complex I	39	37	2	2.84 × 10^−9^	0.81 ± 0.03	2.63 × 10^−8^
Complex II	4	0	4	0.13	1.12 ± 0.03	0.024
Complex III	9	8	1	0.039	0.82 ± 0.03	6.31 × 10^−4^
Complex IV	19	16	3	0.0044	0.83 ± 0.03	8.41 × 10^−6^
Complex V	18	17	1	1.45 × 10^−4^	0.80 ± 0.04	2.25 × 10^−5^
All 5 complexes	89	78	11	1.37 × 10^−13^	0.83 ± 0.02	<2.2 × 10^−16^
TCA cycle	21	7	14	0.189	1.03 ± 0.02	0.171
Protein import complex subunits	25	20	5	0.0041	0.88 ± 0.02	3.29 × 10^−5^
SLC25 family	40	21	19	0.88	1.011 ± 0.02	0.628

## Supplementary Materials

The following are available online at https://www.mdpi.com/1422-0067/22/4/2046/s1, Table S1: Pathways overrepresented amongst downregulated transcripts in ME/CFS lymphoblasts. Table S2. Pathways overrepresented amongst upregulated transcripts in ME/CFS lymphoblasts. Table S3: Transcript levels of CD cell markers and Sirtuins detected in whole cell transcriptomes of ME/CFS and control lymphoblasts. Table S4. Pathways over-represented amongst upregulated proteins in ME/CFS lymphoblasts. Table S5. Pathways over-represented amongst downregulated proteins in ME/CFS lymphoblasts. Table S6: Protein levels of CD cell markers detected in whole cell proteomes of ME/CFS and control lymphoblasts. Figure S1: Western blots and qRT-PCR results verifying whole-cell proteome and transcriptome observations, respectively. (A) ACO2, SDHA and MDH1 expression as measured by semiquantitative Western blotting are consistent with expression as measured by whole-cell proteomics. In each pair of charts, the left panel is the result of semiquantitative western blotting and the right panel is the relative abundance of the same protein in the proteomics. Western blot sample sizes are as follows. ACO2: ME/CFS *n* = 21, Control = 22. SdhA: ME/CFS *n* = 17, Control = 19. MDH1: ME/CFS *n* = 6, Control = 7. Whole-cell proteomics experiment: ME/CFS *n* = 34, Control = 31. (B) GLS, SdhB, NDUFB1 and NDUFB10 transcript levels relative to HIST1H1C were consistent in direction between qRTPCR experiments and the whole-cell transcriptomes. In each pair of charts, the left panel is the result of semiquantitative qRT-PCR and the right panel is the relative abundance of the same transcript in the transcriptomics. GLS qRT-PCR: ME/CFS *n* = 23, Control = 16. SdhB qRTPCR: ME/CFS *n* = 23, Control = 16. NDUFB1 and NDUFB10 GLS qRT-PCR: ME/CFS *n* = 22, Control = 16. Whole-cell transcriptomics experiment: ME/CFS *n* = 23, Control = 17. Figure S2: Example images of confirmatory semi-quantitative Western blots and loading controls (stain-free gels). (A) Example Western blot undertaken as part of the experiments verifying ACO2 levels in the whole-cell proteomes. The intensity of individual bands was digitally quantified and normalized to internal control cell lines included in every experiment. Protein ladder was visible in the stain-free gel but not in this particular corresponding blot. The chart below the blot shows the signal of the band of interest normalized to total protein loading. (B) Example Western blot undertaken as part of the experiments verifying SDHA levels in the whole-cell proteomes. The intensity of individual bands was digitally quantified and normalized to internal control cell lines included in every experiment. The chart below the blot shows the signal of the band of interest normalized to total protein loading. Figure S3: Expression of SLC25A11 and MDH1 is elevated in the whole-cell proteomes of ME/CFS lymphoblasts, while that of BCKDK is unaltered (two-tailed *t* tests in each case). Error bars represent standard error of the mean. Relative protein abundance was obtained from iBAQ values normalised to the control average within the respective individual experiments.
