# Supplementary material for: Dysregulated Provision of Oxidisable Substrates to the Mitochondria in ME/CFS Lymphoblasts
Source: Int J Mol Sci. 2021 Feb 19;22(4):2046. doi: 10.3390/ijms22042046 (PMC7921983; doi:10.3390/ijms22042046)
Supplement: Supplementary file 1 [file ijms-22-02046-s001.pdf]

Supplemental Information

Pathway over-representation analysis of differentially expressed transcripts and proteins in ME/CFS lymphoblasts

Table S1: Pathways overrepresented amongst downregulated transcripts in ME/CFS lymphoblasts.

Reactome pathway	Number of genes			Fold enrichment	Binomial test P-value	False discovery rate (FDR)
	In entire experiment	In downregulated fraction (Q<0.05)	Expected			
Formation of a pool of free 40S subunits (R-HSA-72689)	97	63	9.92	6.35	5.54×10^{-30}	1.20×10^{-26}
GTP hydrolysis and joining of the 60S ribosomal subunit (R-HSA-72706)	107	65	10.94	5.94	2.68×10^{-29}	1.45×10^{-26}
Cap-dependent Translation Initiation (R-HSA-72737)	114	67	11.66	5.75	2.35×10^{-29}	1.69×10^{-26}
Eukaryotic Translation Initiation (R-HSA-72613)	114	67	11.66	5.75	2.35×10^{-29}	2.54×10^{-26}
L13a-mediated translational silencing of Ceruloplasmin expression (R-HSA-156827)	106	64	10.84	5.9	1.01×10^{-28}	3.63×10^{-26}
Viral mRNA Translation (R-HSA-192823)	85	58	8.69	6.67	9.12×10^{-29}	3.94×10^{-26}

Translation (R-HSA-72766)	286	105	29.24	3.59	1.94×10^{-28}	5.98×10^{-26}
Peptide chain elongation (R-HSA-156902)	85	57	8.69	6.56	6.35×10^{-28}	1.71×10^{-25}
Selenocysteine synthesis (R-HSA-2408557)	89	58	9.1	6.37	8.94×10^{-28}	1.93×10^{-25}
Eukaryotic Translation Elongation (R-HSA-156842)	89	58	9.1	6.37	8.94×10^{-28}	2.14×10^{-25}
Regulation of expression of SLITs and ROBOs (R-HSA-9010553)	155	75	15.85	4.73	1.57×10^{-27}	3.08×10^{-25}
Eukaryotic Translation Termination (R-HSA-72764)	89	57	9.1	6.26	5.95×10^{-27}	1.07×10^{-24}
Nonsense Mediated Decay (NMD) independent of the Exon Junction Complex (EJC) (R-HSA-975956)	91	57	9.31	6.13	1.74×10^{-26}	2.89×10^{-24}
Nonsense Mediated Decay (NMD) enhanced by the Exon Junction Complex (EJC) (R-	111	62	11.35	5.46	4.10×10^{-26}	5.89×10^{-24}

HSA-975957)						
Nonsense-Mediated Decay (NMD) (R-HSA-927802)	111	62	11.35	5.46	4.10×10^{-26}	6.31×10^{-24}
Response of EIF2AK4 (GCN2) to amino acid deficiency (R-HSA-9633012)	97	58	9.92	5.85	6.10×10^{-26}	8.22×10^{-24}
SRP-dependent cotranslational protein targeting to membrane (R-HSA-1799339)	108	60	11.04	5.43	3.39×10^{-25}	4.31×10^{-23}
Selenoamino acid metabolism (R-HSA-2408522)	106	59	10.84	5.44	7.75×10^{-25}	9.29×10^{-23}
Metabolism of RNA (R-HSA-8953854)	635	159	64.93	2.45	1.11×10^{-24}	1.26×10^{-22}
rRNA processing (R-HSA-72312)	194	79	19.84	3.98	2.67×10^{-24}	2.88×10^{-22}
Influenza Viral RNA Transcription and Replication (R-HSA-168273)	127	63	12.99	4.85	7.32×10^{-24}	7.52×10^{-22}
rRNA processing in the nucleus and cytosol (R-HSA-8868773)	185	76	18.92	4.02	1.22×10^{-23}	1.19×10^{-21}
Major pathway of rRNA	175	73	17.89	4.08	3.99×10^{-23}	3.74×10^{-21}

processing in the nucleolus and cytosol (R-HSA-6791226)						
Signaling by ROBO receptors (R-HSA-376176)	191	76	19.53	3.89	7.74×10^{-23}	6.96×10^{-21}
Influenza Life Cycle (R-HSA-168255)	136	63	13.91	4.53	2.30×10^{-22}	1.99×10^{-20}
Influenza Infection (R-HSA-168254)	146	64	14.93	4.29	1.71×10^{-21}	1.42×10^{-19}
Metabolism of amino acids and derivatives (R-HSA-71291)	278	89	28.43	3.13	2.06×10^{-20}	1.65×10^{-18}
Infectious disease (R-HSA-5663205)	411	103	42.03	2.45	3.24×10^{-16}	2.50×10^{-14}
Formation of the ternary complex, and subsequently, the 43S complex (R-HSA-72695)	49	31	5.01	6.19	3.65×10^{-15}	2.71×10^{-13}
Translation initiation complex formation (R-HSA-72649)	55	31	5.62	5.51	7.34×10^{-14}	5.11×10^{-12}
Ribosomal scanning and start codon recognition (R-HSA-72702)	55	31	5.62	5.51	7.34×10^{-14}	5.28×10^{-12}
Activation of the	56	31	5.73	5.41	1.17×10^{-13}	7.86×10^{-12}

mRNA upon binding of the cap-binding complex and eIFs, and subsequent binding to 43S (R-HSA-72662)						
Cellular responses to external stimuli (R-HSA-8953897)	486	106	49.7	2.13	7.00×10^{-13}	4.58×10^{-11}
Cellular responses to stress (R-HSA-2262752)	481	105	49.18	2.13	8.61×10^{-13}	5.46×10^{-11}
Axon guidance (R-HSA-422475)	402	91	41.11	2.21	4.76×10^{-12}	2.93×10^{-10}
Respiratory electron transport, ATP synthesis by chemiosmotic coupling, and heat production by uncoupling proteins. (R-HSA-163200)	109	40	11.15	3.59	1.33×10^{-11}	7.95×10^{-10}
Respiratory electron transport (R-HSA-611105)	90	34	9.2	3.69	2.14×10^{-10}	1.25×10^{-8}
The citric acid (TCA) cycle and respiratory electron	152	44	15.54	2.83	1.95×10^{-9}	1.11×10^{-7}

transport (R-HSA-1428517)						
Mitochondrial translation elongation (R-HSA-5389840)	87	31	8.9	3.48	5.03×10^{-9}	2.78×10^{-7}
Metabolism of proteins (R-HSA-392499)	1533	226	156.76	1.44	1.21×10^{-8}	6.54×10^{-7}
Mitochondrial translation initiation (R-HSA-5368286)	87	30	8.9	3.37	1.80×10^{-8}	9.27×10^{-7}
Mitochondrial translation termination (R-HSA-5419276)	87	30	8.9	3.37	1.80×10^{-8}	9.50×10^{-7}
Mitochondrial translation (R-HSA-5368287)	93	31	9.51	3.26	2.23×10^{-8}	1.12×10^{-6}
Metabolism (R-HSA-1430728)	1513	221	154.71	1.43	3.80×10^{-8}	1.86×10^{-6}
Complex I biogenesis (R-HSA-6799198)	49	21	5.01	4.19	7.59×10^{-8}	3.64×10^{-6}
Developmental Biology (R-HSA-1266738)	616	105	62.99	1.67	3.82×10^{-7}	1.79×10^{-5}
Disease (R-HSA-1643685)	863	136	88.25	1.54	5.25×10^{-7}	2.41×10^{-5}
Regulation of mRNA stability by proteins that bind AU-rich elements (R-HSA-450531)	84	25	8.59	2.91	3.57×10^{-6}	1.60×10^{-4}

Protein localization (R-HSA-9609507)	145	34	14.83	2.29	1.19×10^{-5}	5.23×10^{-4}
Vif-mediated degradation of APOBEC3G (R-HSA-180585)	51	17	5.21	3.26	3.16×10^{-5}	0.0014
Negative regulation of NOTCH4 signaling (R-HSA-9604323)	52	17	5.32	3.2	4.00×10^{-5}	0.0017
Metabolism of polyamines (R-HSA-351202)	55	17	5.62	3.02	7.83×10^{-5}	0.0033
mRNA Splicing (R-HSA-72172)	183	37	18.71	1.98	1.07×10^{-4}	0.0042
Autodegradation of Cdh1 by Cdh1:APC/C (R-HSA-174084)	62	18	6.34	2.84	1.06×10^{-4}	0.0042
Oxygen-dependent proline hydroxylation of Hypoxia-inducible Factor Alpha (R-HSA-1234176)	62	18	6.34	2.84	1.06×10^{-4}	0.0043
FBXL7 down-regulates AURKA during mitotic entry and in early mitosis (R-	52	16	5.32	3.01	1.32×10^{-4}	0.0050

HSA-8854050)						
HIV Infection (R-HSA-162906)	213	41	21.78	1.88	1.31×10^{-4}	0.0051
Regulation of activated PAK-2p34 by proteasome mediated degradation (R-HSA-211733)	48	15	4.91	3.06	1.79×10^{-4}	0.0067
APC/C:Cdc20 mediated degradation of Securin (R-HSA-174154)	65	18	6.65	2.71	1.88×10^{-4}	0.0069
Degradation of DVL (R-HSA-4641258)	54	16	5.52	2.9	2.00×10^{-4}	0.0071
mRNA Splicing - Major Pathway (R-HSA-72163)	175	35	17.89	1.96	1.98×10^{-4}	0.0071
Assembly of the pre-replicative complex (R-HSA-68867)	66	18	6.75	2.67	2.25×10^{-4}	0.0077
Regulation of ornithine decarboxylase (ODC) (R-HSA-350562)	49	15	5.01	2.99	2.23×10^{-4}	0.0078
Ubiquitin-dependent degradation of Cyclin D (R-HSA-75815)	50	15	5.11	2.93	2.75×10^{-4}	0.0085
Autodegradation of the E3 ubiquitin	50	15	5.11	2.93	2.75×10^{-4}	0.0086

ligase COP1 (R-HSA-349425)						
Vpu mediated degradation of CD4 (R-HSA-180534)	50	15	5.11	2.93	2.75×10^{-4}	0.0087
Regulation of RAS by GAPs (R-HSA-5658442)	61	17	6.24	2.73	2.60×10^{-4}	0.0088
Ubiquitin Mediated Degradation of Phosphorylated Cdc25A (R-HSA-69601)	50	15	5.11	2.93	2.75×10^{-4}	0.0089
Degradation of GLI2 by the proteasome (R-HSA-5610783)	56	16	5.73	2.79	2.98×10^{-4}	0.0089
p53-Independent DNA Damage Response (R-HSA-69610)	50	15	5.11	2.93	2.75×10^{-4}	0.0090
GLI3 is processed to GLI3R by the proteasome (R-HSA-5610785)	56	16	5.73	2.79	2.98×10^{-4}	0.0090
p53-Independent G1/S DNA damage checkpoint (R-HSA-69613)	50	15	5.11	2.93	2.75×10^{-4}	0.0091
Regulation of RUNX2	62	17	6.34	2.68	3.12×10^{-4}	0.0092

expression and activity (R-HSA-8939902)						
Orc1 removal from chromatin (R-HSA-68949)	68	18	6.95	2.59	3.19×10^{-4}	0.0093
Regulation of Apoptosis (R-HSA-169911)	51	15	5.21	2.88	3.37×10^{-4}	0.0097
Degradation of GLI1 by the proteasome (R-HSA-5610780)	57	16	5.83	2.75	3.60×10^{-4}	0.010
Cdc20:Phospho-APC/C mediated degradation of Cyclin A (R-HSA-174184)	69	18	7.06	2.55	3.78×10^{-4}	0.010
Cellular response to hypoxia (R-HSA-1234174)	69	18	7.06	2.55	3.78×10^{-4}	0.010
Switching of origins to a post-replicative state (R-HSA-69052)	87	21	8.9	2.36	3.58×10^{-4}	0.010
Mitochondrial protein import (R-HSA-1268020)	63	17	6.44	2.64	3.73×10^{-4}	0.010
CDK-mediated phosphorylation and removal of Cdc6 (R-HSA-69017)	69	18	7.06	2.55	3.78×10^{-4}	0.010

AUF1 (hnRNP D0) binds and destabilizes mRNA (R-HSA-450408)	52	15	5.32	2.82	4.11×10^{-4}	0.011
APC:Cdc20 mediated degradation of cell cycle proteins prior to satisfation of the cell cycle checkpoint (R-HSA-179419)	70	18	7.16	2.51	4.46×10^{-4}	0.012
APC/C:Cdh1 mediated degradation of Cdc20 and other APC/C:Cdh1 targeted proteins in late mitosis/early G1 (R-HSA-174178)	70	18	7.16	2.51	4.46×10^{-4}	0.011
Degradation of AXIN (R-HSA-4641257)	53	15	5.42	2.77	4.98×10^{-4}	0.012
Hh mutants that don't undergo autocatalytic processing are degraded by ERAD (R-HSA-5362768)	53	15	5.42	2.77	4.98×10^{-4}	0.012
SCF-beta-TrCP mediated degradation	53	15	5.42	2.77	4.98×10^{-4}	0.013

of Emi1 (R-HSA-174113)						
Regulation of RUNX3 expression and activity (R-HSA-8941858)	53	15	5.42	2.77	4.98×10^{-4}	0.013
Synthesis of DNA (R-HSA-69239)	116	25	11.86	2.11	5.45×10^{-4}	0.013
Processing of Capped Intron-Containing Pre-mRNA (R-HSA-72203)	229	41	23.42	1.75	5.50×10^{-4}	0.013
DNA Replication (R-HSA-69306)	123	26	12.58	2.07	5.65×10^{-4}	0.013
Hh mutants abrogate ligand secretion (R-HSA-5387390)	54	15	5.52	2.72	6.01×10^{-4}	0.014
Stabilization of p53 (R-HSA-69541)	54	15	5.52	2.72	6.01×10^{-4}	0.014
Regulation of PTEN stability and activity (R-HSA-8948751)	66	17	6.75	2.52	6.22×10^{-4}	0.014
APC/C:Cdc20 mediated degradation of mitotic proteins (R-HSA-176409)	72	18	7.36	2.44	6.15×10^{-4}	0.014
Degradation of beta-catenin by the destruction complex	79	19	8.08	2.35	6.94×10^{-4}	0.015

(R-HSA-195253)						
ER-Phagosome pathway (R-HSA-1236974)	79	19	8.08	2.35	6.94×10^{-4}	0.015
Activation of APC/C and APC/C:Cdc20 mediated degradation of mitotic proteins (R-HSA-176814)	73	18	7.46	2.41	7.18×10^{-4}	0.016
Cross-presentation of soluble exogenous antigens (endosomes) (R-HSA-1236978)	44	13	4.5	2.89	7.82×10^{-4}	0.017
NIK-->noncanonical NF-kB signaling (R-HSA-5676590)	57	15	5.83	2.57	0.0010	0.021
The role of GTSE1 in G2/M progression after G2 checkpoint (R-HSA-8852276)	69	17	7.06	2.41	0.0010	0.021
Hedgehog ligand biogenesis (R-HSA-5358346)	57	15	5.83	2.57	0.0010	0.021
CDT1 association with the CDC6:ORC:origin complex (R-HSA-68827)	57	15	5.83	2.57	0.0010	0.022

DNA Replication Pre-Initiation (R-HSA-69002)	82	19	8.38	2.27	0.0011	0.022
UCH proteinases (R-HSA-5689603)	89	20	9.1	2.2	0.0012	0.023
Antigen processing-Cross presentation (R-HSA-1236975)	89	20	9.1	2.2	0.0012	0.023
SCF(Skp2)-mediated degradation of p27/p21 (R-HSA-187577)	58	15	5.93	2.53	0.0012	0.023
Dectin-1 mediated noncanonical NF-kB signaling (R-HSA-5607761)	58	15	5.93	2.53	0.0012	0.024
mRNA Splicing - Minor Pathway (R-HSA-72165)	52	14	5.32	2.63	0.0012	0.024
Defective CFTR causes cystic fibrosis (R-HSA-5678895)	58	15	5.93	2.53	0.0012	0.024
Regulation of APC/C activators between G1/S and early anaphase (R-HSA-176408)	77	18	7.87	2.29	0.0013	0.025
tRNA modification in the	41	12	4.19	2.86	0.0013	0.025

nucleus and cytosol (R-HSA-6782315)						
Asymmetric localization of PCP proteins (R-HSA-4608870)	60	15	6.14	2.44	0.0017	0.031
RAF/MAP kinase cascade (R-HSA-5673001)	162	30	16.57	1.81	0.0018	0.032
RUNX1 regulates transcription of genes involved in differentiation of HSCs (R-HSA-8939236)	80	18	8.18	2.2	0.0019	0.035
KSRP (KHSRP) binds and destabilizes mRNA (R-HSA-450604)	17	7	1.74	4.03	0.0021	0.038
p53-Dependent G1 DNA Damage Response (R-HSA-69563)	62	15	6.34	2.37	0.0023	0.040
p53-Dependent G1/S DNA damage checkpoint (R-HSA-69580)	62	15	6.34	2.37	0.0023	0.041
Signaling by NOTCH4 (R-HSA-9013694)	75	17	7.67	2.22	0.0024	0.041
MAPK1/MAPK3 signaling	167	30	17.08	1.76	0.0027	0.047

(R-HSA-5684996)						
Regulation of mitotic cell cycle (R-HSA-453276)	83	18	8.49	2.12	0.0029	0.048
APC/C-mediated degradation of cell cycle proteins (R-HSA-174143)	83	18	8.49	2.12	0.0029	0.049
PCP/CE pathway (R-HSA-4086400)	83	18	8.49	2.12	0.0029	0.049

Table S1: Pathway over-representation analysis of downregulated transcripts in the whole-cell transcriptome using PANTHER gene expression analysis. “Reactome Pathway” was set as the level of biological granularity. Genes were included in the “downregulated fraction” on the basis of possessing a mean log fold change < 0 in the ME/CFS group versus the control mean, and a Q value < 0.05 according to the Benjamini-Hochberg method applied to P values from the F test. The PANTHER analysis was then applied to the list, testing for over-representation of particular pathways in the list using the binomial test, followed by calculation of the False Discovery Rate (FDR, using the Benjamini-Hochberg method). Output was sorted by statistical significance.

Table S2. Pathways overrepresented amongst upregulated transcripts in ME/CFS lymphoblasts.

Reactome pathway	Number of genes			Fold enrichment	Binomial test P-value
	In entire experiment	In upregulated fraction (Q<0.05)	Expected		
RNA Polymerase II Transcription (R-HSA-73857)	1116	105	74.65	1.41	2.74×10^{-4}
Gene expression (Transcription) (R-HSA-74160)	1234	113	82.55	1.37	4.30×10^{-4}
Generic Transcription Pathway (R-HSA-212436)	997	93	66.69	1.39	8.18×10^{-4}
ZBP1(DAI) mediated induction of type I IFNs (R-HSA-1606322)	20	6	1.34	4.48	0.0025
Cytosolic sensors of pathogen-associated DNA (R-HSA-1834949)	61	11	4.08	2.7	0.0032
RIP-mediated NFkB activation via ZBP1 (R-HSA-1810476)	16	5	1.07	4.67	0.0048
Signaling by MET (R-HSA-6806834)	56	10	3.75	2.67	0.0052
Cargo recognition for clathrin-mediated endocytosis (R-HSA-8856825)	70	11	4.68	2.35	0.0086
Transport of vitamins, nucleosides, and related	27	6	1.81	3.32	0.010

molecules (R-HSA-425397)					
HATs acetylate histones (R-HSA-3214847)	93	13	6.22	2.09	0.011
Antiviral mechanism by IFN-stimulated genes (R-HSA-1169410)	75	11	5.02	2.19	0.014
Netrin-1 signaling (R-HSA-373752)	29	6	1.94	3.09	0.014
Interferon Signaling (R-HSA-913531)	162	19	10.84	1.75	0.015
p75NTR signals via NF-kB (R-HSA-193639)	14	4	0.94	4.27	0.015
Netrin mediated repulsion signals (R-HSA-418886)	3	2	0.2	9.97	0.018
Metabolism of ingested H2SeO4 and H2SeO3 into H2Se (R-HSA-2408550)	3	2	0.2	9.97	0.018
Transport of nucleotide sugars (R-HSA-727802)	9	3	0.6	4.98	0.023
OAS antiviral response (R-HSA-8983711)	9	3	0.6	4.98	0.023
STING mediated induction of host immune responses (R-HSA-1834941)	16	4	1.07	3.74	0.024
Signaling by Hippo (R-	16	4	1.07	3.74	0.024

HSA-2028269)					
Regulation of cholesterol biosynthesis by SREBP (SREBF) (R-HSA-1655829)	52	8	3.48	2.3	0.026
Downstream signal transduction (R-HSA-186763)	25	5	1.67	2.99	0.028
PPARA activates gene expression (R-HSA-1989781)	95	12	6.35	1.89	0.029
Clathrin-mediated endocytosis (R-HSA-8856828)	106	13	7.09	1.83	0.029
NR1H2 & NR1H3 regulate gene expression linked to triglyceride lipolysis in adipose (R-HSA-9031528)	4	2	0.27	7.47	0.030
Transport of organic anions (R-HSA-879518)	4	2	0.27	7.47	0.030
RHO GTPases activate KTN1 (R-HSA-5625970)	10	3	0.67	4.48	0.030
Regulation of lipid metabolism by PPARalpha (R-HSA-400206)	96	12	6.42	1.87	0.031
Toll Like Receptor 9 (TLR9) Cascade (R-HSA-168138)	86	11	5.75	1.91	0.033

CREB1 phosphorylation through NMDA receptor-mediated activation of RAS signaling (R-HSA-442742)	18	4	1.2	3.32	0.034
Nuclear Receptor transcription pathway (R-HSA-383280)	27	5	1.81	2.77	0.037
Transport of small molecules (R-HSA-382551)	443	40	29.63	1.35	0.037
N-Glycan antennae elongation (R-HSA-975577)	11	3	0.74	4.08	0.039
p75NTR recruits signalling complexes (R-HSA-209543)	11	3	0.74	4.08	0.039
PECAM1 interactions (R-HSA-210990)	11	3	0.74	4.08	0.039
TRIF(TICAM1)-mediated TLR4 signaling (R-HSA-937061)	89	11	5.95	1.85	0.040
MyD88-independent TLR4 cascade (R-HSA-166166)	89	11	5.95	1.85	0.040
Retrograde transport at the Trans-Golgi-Network (R-HSA-6811440)	47	7	3.14	2.23	0.041
Membrane Trafficking (R-HSA-199991)	526	46	35.19	1.31	0.042
Transport to the Golgi and	159	17	10.64	1.6	0.043

subsequent modification (R-HSA-948021)					
Vesicle-mediated transport (R-HSA-5653656)	540	47	36.12	1.3	0.043
NR1H2 & NR1H3 regulate gene expression to limit cholesterol uptake (R-HSA-9031525)	5	2	0.33	5.98	0.045
Signaling by Receptor Tyrosine Kinases (R-HSA-9006934)	334	31	22.34	1.39	0.045
Signaling by NTRKs (R-HSA-166520)	80	10	5.35	1.87	0.046
Diseases of Immune System (R-HSA-5260271)	20	4	1.34	2.99	0.047
Diseases associated with the TLR signaling cascade (R-HSA-5602358)	20	4	1.34	2.99	0.047
SLC transporter disorders (R-HSA-5619102)	59	8	3.95	2.03	0.048
Gastrin-CREB signalling pathway via PKC and MAPK (R-HSA-881907)	12	3	0.8	3.74	0.048
Ion channel transport (R-HSA-983712)	103	12	6.89	1.74	0.048

TRAF6 mediated induction of NFkB and MAP kinases upon TLR7/8 or 9 activation (R-HSA-975138)	81	10	5.42	1.85	0.049
Activation of gene expression by SREBF (SREBP) (R-HSA-2426168)	39	6	2.61	2.3	0.049

Table S2: Pathway over-representation analysis of upregulated transcripts in whole-cell transcriptome data using PANTHER gene expression analysis. “Reactome Pathway” was set as the level of biological granularity. Genes were included in the “upregulated fraction” on the basis of possessing a mean log fold change > 0 in the ME/CFS group versus the control mean, and a Q value < 0.05 according to the Benjamini-Hochberg method applied to P values from the F test. Output was sorted according to statistical significance. The PANTHER analysis was then applied to the list testing for over-representation of particular pathways in the list using the binomial test. The FDR correction was not applied to the PANTHER analysis. Output was sorted by statistical significance.

Table S3: Transcript levels of CD cell markers and Sirtuins detected in whole cell transcriptomes of ME/CFS and control lymphoblasts.

Gene Name	LogFC	P-value	Q-value
CD151	-0.13	0.51	1.71
CD164	0.28	0.028	0.032
CD180	-0.007	0.98	95.21
CD19	-0.42	0.0007	0.00074
CD200	-0.085	0.80	6.79
CD22	0.051	0.80	6.72
CD226	0.24	0.38	0.94
CD24	0.38	0.31	0.68
CD27	-0.33	0.22	0.40
CD274	0.16	0.51	1.70
CD2AP	0.11	0.24	0.45
CD300A	0.31	0.13	0.19
CD320	-0.37	0.052	0.065
CD37	-0.12	0.33	0.73
CD38	-0.15	0.55	1.96
CD40	0.079	0.52	1.79
CD44	-0.053	0.75	5.08
CD46	0.19	0.21	0.38
CD47	-0.33	0.031	0.035
CD48	0.10	0.56	2.13
CD52	-0.39	0.030	0.035
CD53	0.24	0.057	0.072
CD55	0.30	0.17	0.27
CD58	0.026	0.85	9.64
CD59	-0.13	0.27	0.54
CD63	-0.33	0.056	0.070
CD69	0.20	0.32	0.71
CD70	-0.00068	1.00	341.15
CD72	0.020	0.94	25.35
CD74	0.069	0.67	3.41
CD79A	-0.34	0.040	0.048
CD79B	-0.25	0.16	0.26

CD80	0.047	0.82	7.79
CD81	-0.15	0.22	0.39
CD82	0.17	0.29	0.61
CD83	0.23	0.22	0.40
CD84	0.053	0.82	7.99
CD86	-0.15	0.41	1.07
CD99	0.045	0.79	6.66
CD99L2	-0.23	0.14	0.21
SIRT1	0.29	0.091	0.13
SIRT2	0.23	0.0058	0.0060
SIRT3	-0.22	0.011	0.012
SIRT4	0.042	0.86	10.50
SIRT5	-0.11	0.18	0.31
SIRT6	-0.30	0.012	0.013
SIRT7	-0.080	0.31	0.68

Table S3: Transcript levels of CD cell markers and Sirtuins detected in whole cell transcriptomics of ME/CFS and control lymphoblasts. LogFC represents the log fold change in the ME/CFS group relative to the control group. Q values were assigned according to the Benjamini-Hochberg method.

Table S4. Pathways over-represented amongst upregulated proteins in ME/CFS lymphoblasts.

Reactome pathway	Number of proteins: In entire dataset	Number of proteins: In significantly upregulated fraction (Q<0.05)	Number of proteins: Expected	Fold enrichment	Binomial test P value
Metabolism (R-HSA-1430728)	789	66	43.61	1.51	2.12×10^{-4}
Mitochondrial Fatty Acid Beta-Oxidation (R-HSA-77289)	23	7	1.27	5.51	3.32×10^{-4}
Interferon gamma signaling (R-HSA-877300)	49	10	2.71	3.69	4.60×10^{-4}
Beta oxidation of palmitoyl-CoA to myristoyl-CoA (R-HSA-77305)	3	3	0.17	18.09	6.64×10^{-4}
Mitochondrial fatty acid beta-oxidation of saturated fatty acids (R-HSA-77286)	7	4	0.39	10.34	6.72×10^{-4}
Phosphorylation of CD3 and TCR zeta chains (R-HSA-202427)	13	5	0.72	6.96	8.53×10^{-4}
Mitochondrial calcium ion transport (R-HSA-8949215)	13	5	0.72	6.96	8.53×10^{-4}
Beta oxidation of lauroyl-CoA to decanoyl-CoA-CoA (R-HSA-77310)	4	3	0.22	13.57	0.0015
Glutamate Neurotransmitter Release Cycle (R-HSA-210500)	4	3	0.22	13.57	0.0015
Interleukin-2 family signaling (R-HSA-451927)	15	5	0.83	6.03	0.0016
PD-1 signaling (R-HSA-389948)	15	5	0.83	6.03	0.0016
Cytokine Signaling in Immune system (R-HSA-1280215)	327	31	18.08	1.72	0.0023
Interleukin-9 signaling (R-HSA-8985947)	5	3	0.28	10.85	0.0028

Beta oxidation of hexanoyl-CoA to butanoyl-CoA (R-HSA-77350)	5	3	0.28	10.85	0.0028
Beta oxidation of octanoyl-CoA to hexanoyl-CoA (R-HSA-77348)	5	3	0.28	10.85	0.0028
Beta oxidation of decanoyl-CoA to octanoyl-CoA-CoA (R-HSA-77346)	5	3	0.28	10.85	0.0028
Interleukin-21 signaling (R-HSA-9020958)	5	3	0.28	10.85	0.0028
Mitochondrial fatty acid beta-oxidation of unsaturated fatty acids (R-HSA-77288)	5	3	0.28	10.85	0.0028
Fatty acid metabolism (R-HSA-8978868)	63	10	3.48	2.87	0.0029
Translocation of ZAP-70 to Immunological synapse (R-HSA-202430)	11	4	0.61	6.58	0.0035
Generation of second messenger molecules (R-HSA-202433)	18	5	0.99	5.03	0.0035
Cell-Cell communication (R-HSA-1500931)	27	6	1.49	4.02	0.0042
Interleukin-2 signaling (R-HSA-9020558)	6	3	0.33	9.05	0.0047
Acyl chain remodeling of CL (R-HSA-1482798)	2	2	0.11	18.09	0.0057
Adenylate cyclase inhibitory pathway (R-HSA-170670)	2	2	0.11	18.09	0.0057
Beta oxidation of myristoyl-CoA to lauroyl-CoA (R-HSA-77285)	2	2	0.11	18.09	0.0057
Signaling by PDGF (R-HSA-186797)	13	4	0.72	5.57	0.0062
Downstream signal transduction (R-HSA-186763)	13	4	0.72	5.57	0.0062

Nephrin family interactions (R-HSA-373753)	7	3	0.39	7.75	0.0072
Signaling by cytosolic FGFR1 fusion mutants (R-HSA-1839117)	8	3	0.44	6.78	0.010
Processing of SMDT1 (R-HSA-8949664)	8	3	0.44	6.78	0.010
Interleukin-20 family signaling (R-HSA-8854691)	8	3	0.44	6.78	0.010
Erythropoietin activates STAT5 (R-HSA-9027283)	3	2	0.17	12.06	0.012
Release of apoptotic factors from the mitochondria (R-HSA-111457)	3	2	0.17	12.06	0.012
STAT5 Activation (R-HSA-9645135)	3	2	0.17	12.06	0.012
Mitochondrial biogenesis (R-HSA-1592230)	45	7	2.49	2.81	0.013
Organelle biogenesis and maintenance (R-HSA-1852241)	104	12	5.75	2.09	0.014
FGFR1 mutant receptor activation (R-HSA-1839124)	9	3	0.5	6.03	0.014
Neurotransmitter release cycle (R-HSA-112310)	9	3	0.5	6.03	0.014
Growth hormone receptor signaling (R-HSA-982772)	9	3	0.5	6.03	0.014
TCR signaling (R-HSA-202403)	80	10	4.42	2.26	0.014
Immune System (R-HSA-168256)	805	58	44.5	1.3	0.017
Transmission across Chemical Synapses (R-HSA-112315)	60	8	3.32	2.41	0.019
Metabolism of amino acids and derivatives (R-HSA-71291)	220	20	12.16	1.64	0.021
Signaling by Leptin (R-HSA-2586552)	4	2	0.22	9.05	0.021
Gluconeogenesis (R-HSA-70263)	19	4	1.05	3.81	0.022

Signaling by FGFR1 in disease (R-HSA-5655302)	11	3	0.61	4.93	0.024
EPH-Ephrin signaling (R-HSA-2682334)	40	6	2.21	2.71	0.025
The citric acid (TCA) cycle and respiratory electron transport (R-HSA-1428517)	115	12	6.36	1.89	0.027
Formation of ATP by chemiosmotic coupling (R-HSA-163210)	12	3	0.66	4.52	0.030
Pentose phosphate pathway (R-HSA-71336)	12	3	0.66	4.52	0.030
RAB geranylgeranylation (R-HSA-8873719)	31	5	1.71	2.92	0.030
Transcriptional activation of mitochondrial biogenesis (R-HSA-2151201)	21	4	1.16	3.45	0.030
Activation of GABAB receptors (R-HSA-991365)	5	2	0.28	7.24	0.032
VxPx cargo-targeting to cilium (R-HSA-5620916)	5	2	0.28	7.24	0.032
Interleukin-15 signaling (R-HSA-8983432)	5	2	0.28	7.24	0.032
ADP signalling through P2Y purinoceptor 12 (R-HSA-392170)	5	2	0.28	7.24	0.032
Sulfide oxidation to sulfate (R-HSA-1614517)	5	2	0.28	7.24	0.032
G-protein activation (R-HSA-202040)	5	2	0.28	7.24	0.032
Interaction between L1 and Ankyrins (R-HSA-445095)	5	2	0.28	7.24	0.032
Aspartate and asparagine metabolism (R-HSA-8963693)	5	2	0.28	7.24	0.032
Phenylalanine and tyrosine metabolism (R-HSA-8963691)	5	2	0.28	7.24	0.032

GABA B receptor activation (R-HSA-977444)	5	2	0.28	7.24	0.032
GABA receptor activation (R-HSA-977443)	5	2	0.28	7.24	0.032
Interferon Signaling (R-HSA-913531)	121	12	6.69	1.79	0.038
Detoxification of Reactive Oxygen Species (R-HSA-3299685)	23	4	1.27	3.15	0.040
Costimulation by the CD28 family (R-HSA-388841)	34	5	1.88	2.66	0.042
FLT3 Signaling (R-HSA-9607240)	96	10	5.31	1.88	0.042
Cargo trafficking to the periciliary membrane (R-HSA-5620920)	14	3	0.77	3.88	0.043
Signaling by SCF-KIT (R-HSA-1433557)	14	3	0.77	3.88	0.043
Prolactin receptor signaling (R-HSA-1170546)	6	2	0.33	6.03	0.044
Nef Mediated CD8 Down-regulation (R-HSA-182218)	6	2	0.33	6.03	0.044
Lysine catabolism (R-HSA-71064)	6	2	0.33	6.03	0.044
Clathrin-mediated endocytosis (R-HSA-8856828)	71	8	3.92	2.04	0.045
Downstream TCR signaling (R-HSA-202424)	71	8	3.92	2.04	0.045
Neuronal System (R-HSA-112316)	71	8	3.92	2.04	0.045
Diseases of metabolism (R-HSA-5668914)	24	4	1.33	3.02	0.045

Table S4: Pathway over-representation analysis of upregulated whole-cell proteome data using PANTHER gene expression analysis. “Reactome Pathway” was set as the level of biological granularity. Proteins were included in the “upregulated fraction” on the basis of possessing a mean fold change > 1 in the ME/CFS group versus the control mean, and a Q value < 0.05 according to the Benjamini-Hochberg method applied to P-values from the t test. The PANTHER analysis was then applied to the list testing for over-representation of particular pathways in the list using the binomial test. The FDR correction was not applied to the PANTHER analysis as it obscured true positives confirmed by alternative investigation of concerned functional groups, including respirometry, western blotting and closer proteome analysis. Output was sorted by statistical significance.

Table S5. Pathways over-represented amongst downregulated proteins in ME/CFS lymphoblasts.

Reactome pathway	Number of proteins: In entire dataset	In significantly downregulated fraction (Q<0.05)	Expected	Fold enrichment	Binomial test P-value
Vitamin B1 (thiamin) metabolism (R-HSA-196819)	1	1	0.01	> 100	0.0097
Defective PMM2 causes PMM2-CDG (CDG-1a) (R-HSA-4043911)	1	1	0.01	> 100	0.0097
Regulation of gene expression by Hypoxia-inducible Factor (R-HSA-1234158)	2	1	0.02	51.26	0.019
Protein repair (R-HSA-5676934)	2	1	0.02	51.26	0.019
RHO GTPases activate PKNs (R-HSA-5625740)	27	2	0.26	7.59	0.029
NOTCH4 Activation and Transmission of Signal to the Nucleus (R-HSA-9013700)	3	1	0.03	34.17	0.029
ALKBH3 mediated reversal of alkylation damage (R-HSA-112126)	3	1	0.03	34.17	0.029
GP1b-IX-V activation signalling (R-HSA-430116)	3	1	0.03	34.17	0.029
Meiosis (R-HSA-1500620)	29	2	0.28	7.07	0.033
Synthesis of GDP-mannose (R-HSA-446205)	4	1	0.04	25.63	0.038
Reproduction (R-HSA-1474165)	32	2	0.31	6.41	0.039
Reversal of alkylation damage by DNA dioxygenases (R-HSA-73943)	5	1	0.05	20.5	0.048

Table S5: Pathway over-representation analysis of downregulated whole-cell proteome data using PANTHER gene expression analysis. "Reactome Pathway" was set as the level of biological granularity. Proteins were included in the "downregulated fraction" on the basis of possessing a mean fold change < 1 in the ME/CFS group versus the control mean, and a Q value < 0.05 according to the Benjamini-Hochberg method applied to P-values from the t test. The PANTHER analysis was then applied to the list, testing for over-representation of particular pathways in the list using binomial test. The FDR correction was not applied to the PANTHER analysis as it obscured true positives confirmed by alternative investigation of concerned functional groups, including respirometry, western blotting and closer proteome analysis. Output was sorted by statistical significance.

Table S6: Protein levels of CD cell markers detected in whole cell proteomes of ME/CFS and control lymphoblasts.

Protein Name	Fold change	P-value	Q-value
CD226	4.06	0.038	0.041
CD2AP	0.95	0.67	2.48
CD37	1.62	0.12	0.15
CD38	1.42	0.11	0.13
CD40	1.22	0.31	0.52
CD44	1.18	0.17	0.23
CD48	1.30	0.016	0.016
CD59	1.04	0.82	5.66
CD70	1.83	0.0010	0.0010
CD74	1.12	0.29	0.45
CD79A	0.56	0.39	0.75
CD81	1.11	0.68	2.62
CD97	1.20	0.39	0.74

Table S6: Protein levels of CD cell markers and Sirtuins detected in whole cell proteomics of ME/CFS and control lymphoblasts. Fold change represents the mean fold change in the ME/CFS group relative to the control group. Q values were assigned according to the Benjamini-Hochberg method.

Figure S1: Verification of proteomics and transcriptomics data.

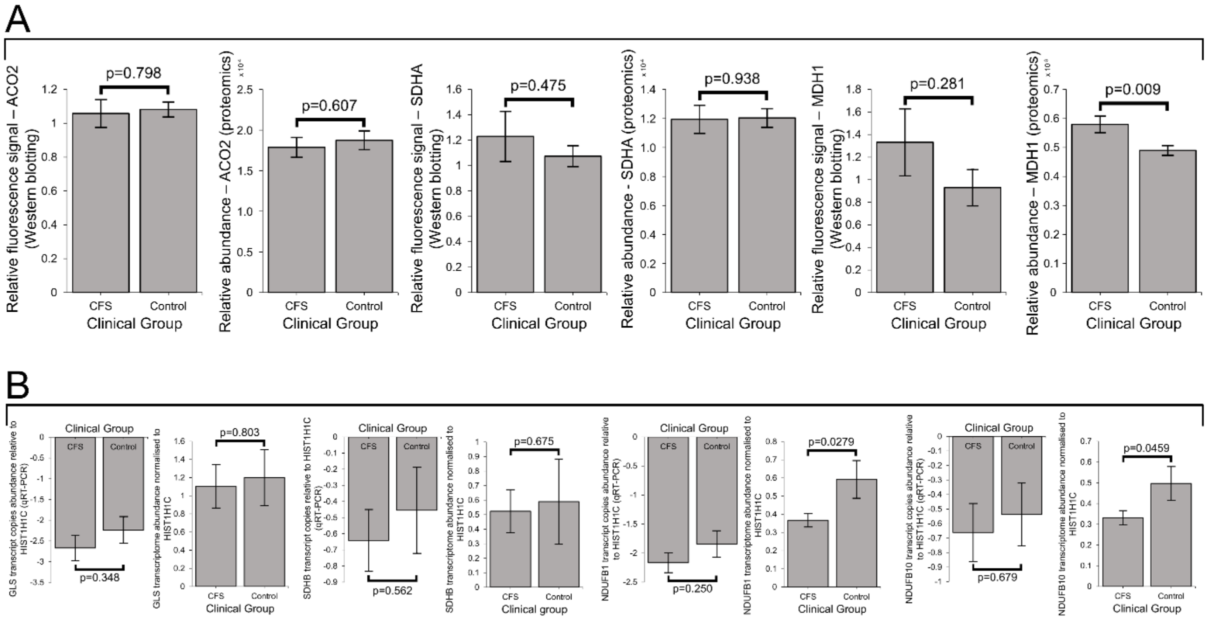

Figure S1: Western blots and qRT-PCR results verifying whole-cell proteome and transcriptome observations, respectively. **(A)** ACO2, SDHA and MDH1 expression as measured by semiquantitative Western blotting are consistent with expression as measured by whole-cell proteomics. In each pair of charts, the left panel is the result of semiquantitative western blotting and the right panel is the relative abundance of the same protein in the proteomics. Western blot sample sizes are as follows. ACO2: ME/CFS n = 21, Control = 22. SdhA: ME/CFS n = 17, Control = 19. MDH1: ME/CFS n = 6, Control = 7. Whole-cell proteomics experiment: ME/CFS n = 34, Control = 31. **(B)** GLS, SdhB, NDUFB1 and NDUFB10 transcript levels relative to HIST1H1C were consistent in direction between qRT-PCR experiments and the whole-cell transcriptomes. In each pair of charts, the left panel is the result of semiquantitative qRT-PCR and the right panel is the relative abundance of the same transcript in the transcriptomics. GLS qRT-PCR: ME/CFS n = 23, Control = 16. SdhB qRT-PCR: ME/CFS n = 23, Control = 16. NDUFB1 and NDUFB10 GLS qRT-PCR: ME/CFS n = 22, Control = 16. Whole-cell transcriptomics experiment: ME/CFS n = 23, Control = 17.

Figure S2. Example semi-quantitative Western blot and loading control images as part of confirmatory experiments verifying the whole-cell proteomics data

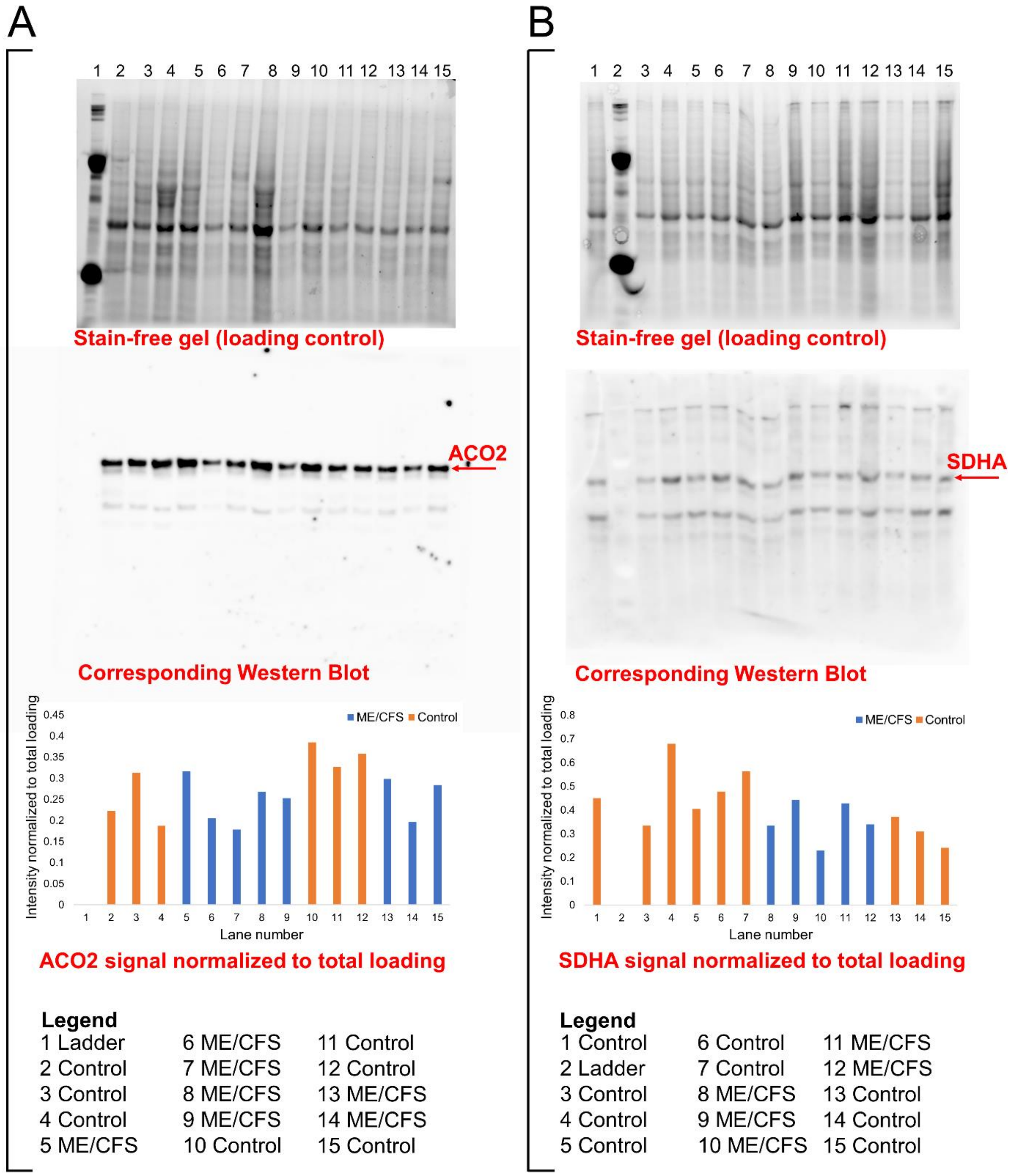

Figure S2: Example images of confirmatory semi-quantitative Western blots and loading controls (stain-free gels). **(A)** Example Western blot undertaken as part of the experiments verifying ACO2 levels in the whole-cell proteomes. The intensity of individual bands was digitally quantified and normalized to internal control cell lines included in every experiment. Protein ladder was visible in the stain-free gel but not in this particular corresponding blot. The chart below the blot shows the signal of the band of interest normalized to total protein loading. **(B)** Example Western blot undertaken as part of the experiments verifying SDHA levels in the whole-cell proteomes. The intensity of individual bands was digitally quantified and

normalized to internal control cell lines included in every experiment. The chart below the blot shows the signal of the band of interest normalized to total protein loading.

Figure S3. SLC25A11 and MDH1 expression are elevated, while that of BCKDK is unaltered in ME/CFS lymphoblasts.

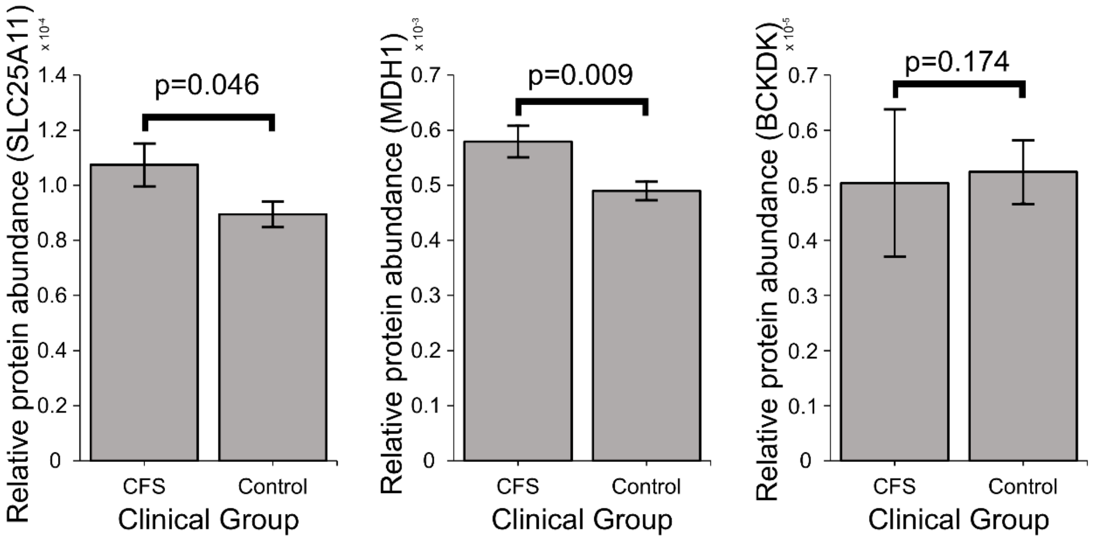

Figure S3: Expression of SLC25A11 and MDH1 is elevated in the whole-cell proteomes of ME/CFS lymphoblasts, while that of BCKDK is unaltered (two-tailed *t* tests in each case). Error bars represent standard error of the mean. Relative protein abundance was obtained from iBAQ values normalised to the control average within the respective individual experiments.
